# Disease, Drought, and Warming: A Triple Threat to a Declining High‐Elevation Amphibian

**DOI:** 10.1002/ece3.73767

**Published:** 2026-06-04

**Authors:** Amanda M. Kissel, L. Mae Lacey, Viorel D. Popescu, Marissa A. Dyck, Larissa L. Bailey, Erin Muths

**Affiliations:** ^1^ U.S. Geological Survey, Fort Collins Science Center Fort Collins Colorado USA; ^2^ Conservation Science Partners Truckee California USA; ^3^ Department of Ecology, Evolution and Environmental Biology, School of Professional Studies Columbia University New York New York USA; ^4^ School of Environmental Studies University of Victoria Victoria British Columbia Canada; ^5^ Department of Fish, Wildlife and Conservation Biology, Graduate Degree Program in Ecology Colorado State University Fort Collins Colorado USA

## Abstract

Managing species in an uncertain future is a reality for natural resource decision makers. Climate change is expected to exacerbate threats such as habitat loss and disease, and cause phenological mismatches, but there is uncertainty in the magnitude of these effects. Amphibians are among the most threatened taxa on earth, and most species in North America are uniquely tied to water availability for breeding, larval development, thermal refugia, and food availability. Changes in water availability and temperature may result in phenological mismatches with one or more of these processes. Thus, quantifying the dependency of amphibians to water on the landscape is critical to understanding how species may respond, as well as understanding the interplay with other threats, such as disease. We developed a dynamic co‐occurrence occupancy model to explore the effects of climate change on the breeding occurrence of boreal toads ( *Anaxyrus boreas* ) and the amphibian chytrid fungus (*Batrachochytrium dendrobatidis*, Bd) in the southern Rocky Mountains (SRM). We derived novel covariates to test hypotheses related to multi‐generational impacts of climate on the dynamics of both boreal toad breeding and Bd. We report estimates of current (2001–2019) and future (2055–2069) occupancy under a range of plausible climate scenarios. The probability of boreal toad breeding occurrence at a site in the SRM declined > 40% from 2001 to 2019, and further declines are likely under future scenarios, particularly as active season length increases. To help integrate this information into management, we developed a web‐based decision support tool to summarize predicted future hydrological and occupancy conditions.

## Graphical abstract

We developed a dynamic co‐occurrence occupancy model to explore the effects of climate change on the occupancy of boreal toads ( *Anaxyrus boreas* ) and the amphibian chytrid fungus (*Batrachochytrium dendrobatidis*, Bd) in the southern Rocky Mountains. We derived novel covariates to test hypotheses related to multi‐generational impacts of climate on the dynamics of both boreal toads and Bd. We found that the probability of boreal toads occupying a site in the southern Rocky Mountains declined > 40% from 2001 to 2019, and further declines are likely under future scenarios.

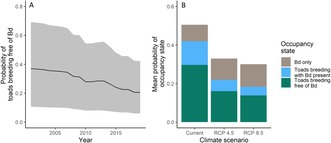

## 1 Introduction

Conservation efforts aimed at maintaining focal species on a landscape typically focus on responding to immediate, perceived threats. However, threats such as disease or drought can be exacerbated with changes in climate. To maximize the effectiveness of management, predictions about species' response to threats and how those responses might vary under different climate scenarios are needed (Mantyka‐Pringle et al. 2012). Predictive assessments are particularly important for amphibians, one of the most imperiled and under‐studied taxa in the world (Scheele et al. 2019), with declines occurring even in protected landscapes (Adams et al. 2013; Kissel, Watry, et al. 2025). Mechanisms and severity of declines vary and depend on species' traits, evolutionary history, local conditions, and susceptibility to disease (Grant et al. 2016; Hof et al. 2011; Hoffmann et al. 2010; Miller et al. 2018).

Nearly all identified mechanisms of amphibian decline are affected by climate change. For example, there is evidence that the spread of amphibian diseases may be exacerbated by drought (McDevitt‐Galles et al. 2022; Muths and Hossack 2022). Changes in stream flows, rainfall patterns, or multi‐year drought can affect the availability of amphibian habitat, leading to declines in occupancy (Irving et al. 2025). In particular, the timing and availability of aquatic breeding habitat are becoming more uncertain, which has demographic consequences for amphibians (Green 2017; Greenberg et al. 2017; Kissel et al. 2024; Nagel et al. 2021; Thompson and Popescu 2021; Todd et al. 2010). For example, increases in the ephemerality of breeding sites may result in sites drying before larvae can metamorphose (Irving et al. 2025). Changes to terrestrial conditions, such as increasing temperatures coupled with reduced water availability, have the potential to exceed the physiological limits of terrestrial amphibians (Greenberg and Palen 2021; Hoffmann et al. 2021; Lertzman‐Lepofsky et al. 2020; Watling and Braga 2015). Additionally, changes in temperature and hydrology can alter the timing of insect metamorphosis, affecting food resources for terrestrial amphibians (Leathers et al. 2024; Nash et al. 2021; Sánchez‐Hernández 2025).

Evidence suggests that drought and increased temperatures can also influence dynamics between amphibians and the amphibian chytrid fungus (*Batrachochytrium dendrobatidis* [Bd]) (Crockett et al. 2020; McDevitt‐Galles et al. 2022; Murphy et al. 2011), which has caused declines globally (Scheele et al. 2019). Declines related to Bd have included several amphibian species in the western United States, and high‐elevation species in particular (Adams et al. 2017; Muths et al. 2003; Rachowicz et al. 2006). Waterborne Bd zoospores embed in the skin of amphibians where they encyst, form zoosporangia in which new zoospores are formed, and then release zoospores, damaging the skin (Berger et al. 2005). Multiple, sympatric amphibian species can serve as Bd hosts, although species may have differential responses (Hulting et al. 2022; Russell et al. 2019; Voyles et al. 2018), and there is evidence that Bd can persist on other, non‐amphibian species, as well as in abiotic conditions (e.g., moist soil, water) (Garmyn et al. 2012; Johnson and Speare 2005; Mosher, Huyvaert, and Bailey 2018; Walker et al. 2007). Although Bd has been shown to persist in cold temperatures, a recent review of experimental and field studies indicated that the optimal growth for Bd was between ~17°C and 25°C (Haver et al. 2022). Thus, it is critical to understand how changes in abiotic factors, such as water availability and temperature, affect amphibians at the individual and population levels and to consider how these factors may additively or synergistically interact with other threats, such as Bd. This knowledge can contribute to landscape‐level management strategies aimed at facilitating amphibian persistence (Kissel et al. 2020).

Landscape‐level habitat data have rarely been used to examine how changes in the environment may be affecting demographic or occupancy rates, because data are seldom available at the necessary temporal and spatial timescales (but refer to Cayuela et al. 2021; Muths et al. 2017). However, advances in remote sensing technologies have increased our capacity to reconstruct landscape‐level data for the recent past (Halabisky et al. 2016; Kissel et al. 2020), expanding our ability to assess how changes in habitat impact amphibians and other wetland‐obligate species (Cartwright et al. 2022; Kissel et al. 2024; Pilliod et al. 2015; Ryan et al. 2014).

Boreal toads ( *Anaxyrus boreas boreas* ) are a species of high elevation amphibian (typically found at > 2000 m) and are likely to be affected by climate change in addition to current threats including Bd. In Colorado, New Mexico, and Wyoming this species is categorized as “Species of Greatest Conservation Need” due to Bd driven declines (Crockett 2023; Muths et al. 2003; Pilliod et al. 2010; Russell et al. 2019). To stem declines and guide recovery, the Boreal Toad Conservation Team (BTCT) was established in 1995 and is led by Colorado Parks and Wildlife, with participation by the states of Colorado, New Mexico, and Wyoming, in addition to multiple federal agencies, universities, and non‐governmental entities (Crockett 2023). Intensive data collection and modeling exercises have been conducted to address widespread declines of the species in the southern Rocky Mountain (SRM) region, including a decision analytic tool to weigh tradeoffs in management strategies (e.g., translocation efforts, habitat manipulations, measures to prevent spread of disease) (Converse et al. 2017; Gerber et al. 2018; Mosher, Bailey, Hubbard, and Huyvaert 2018; Mosher, Bailey, Muths, and Huyvaert 2018; Mosher, Huyvaert, and Bailey 2018). However, these efforts were focused largely on disease and did not consider how climate or other site‐specific covariates may be acting synergistically to affect the persistence of boreal toads. We build on previously developed occupancy models that used ten years of detection/non‐detection data (Gerber et al. 2018; Mosher, Bailey, Hubbard, and Huyvaert 2018; Mosher, Bailey, Muths, and Huyvaert 2018; Mosher, Huyvaert, and Bailey 2018) by adding 10 additional years of data for both boreal toads and Bd, and by incorporating climate covariates hypothesized to affect demographic rates of both boreal toads and Bd into a multi‐species dynamic occupancy model. We developed and tested hypotheses regarding (1) drivers of change in demographic parameters of boreal toads that would be reflected as changes in occupancy (i.e., local colonization and extinction), and (2) occupancy of Bd over time that may also affect boreal toad occupancy (Table 1). We used this model to predict changes in occupancy dynamics under current and future climate conditions.

**TABLE 1:** Hypotheses for each covariate as they relate to each species and parameter.

Parameter	Species	Covariate	Hypothesis
Initial Occupancy Probability	Breeding toads	Proportion of years dried (1985–2000)	*Quadratic effect (convex curve)*—Toads generally breed at perennial or semi‐permanent sites. Sites that dry too frequently prior to toad metamorphosis may result in sustained failed recruitment and a lower probability of occupancy. Thus, we hypothesize that initial occupancy will be low at sites with a high proportion of years dried (representing sites that dry too frequently to sustain toad populations) and occupancy will also be low at sites that rarely dry (representing permanent sites that never dry)
	Bd	Proportion of years dried (1985–2000)	*Negative effect—*Bd requires water to persist long‐term, therefore sites with a higher proportion of years dried are less likely to be occupied by Bd
		Mean annual Bd growing degree days (1985–2000)	*Positive effect—*A higher number of growing degree days represents more days when the temperature is in the ideal thermal range for Bd growth and persistence
Colonization Probability	Breeding toads	Proportion of years dried	*Quadratic effect (convex curve)—*Toads generally breed at perennial or semi‐permanent sites. Sites that dry too frequently prior to toad metamorphosis may result in sustained failed recruitment and a lower probability of occupancy. Thus, we hypothesize that colonization will be low at sites with a high proportion of years dried (representing sites that dry too frequently to sustain toads) and colonization will also be low at sites that rarely dry (representing permanent sites that never dry)
	Bd	Proportion of years dried	*Negative effect—*Bd requires water long‐term to persist, therefore sites with a higher proportion of years dried are less likely to be occupied by Bd
		Bd growing degree days	*Positive effect*—A higher number of growing degree days represents more days when the temperature is in the ideal thermal range for Bd growth and persistence
		Presence of toads	*Positive effect—*Toads are a Bd host, therefore they facilitate colonization
Extinction Probability	Breeding toads	Active season length	*Linear effect—*Very long active seasons may result in higher extinction probability because (1) potential phenological mismatch between foraging resources (i.e., food may not be available late in the season) and toad activity, and (2) increased desiccation risk for all life stages as snow‐dependent (i.e., ephemeral) wetlands are more likely to dry before toads hibernate. Thus, we predict that sites with longer than average active seasons will have higher extinction probabilities
		May cold events	*Positive effect—*As the proportion of years with cold days during the breeding season increases, breeding failure is more likely (temperatures are below the thermal tolerance of eggs and tadpoles) leading to local extinction.
		Proportion of years dried	*Quadratic effect (concave curve)—*Toads generally breed at perennial or semi‐permanent sites. Sites that dry too frequently prior to toad metamorphosis may result in sustained failed recruitment and a lower probability of occupancy and higher local extinction. Thus, we hypothesize that extinction probability will be high at sites with a higher proportion of years dried (representing sites that dry too frequently to sustain toads) and extinction probability will also be high at sites that rarely dry (representing permanent sites that never dry)
		Presence of Bd	*Positive effect* – Bd reduces survival in toads in the southern Rocky Mountains. Therefore, we hypothesize that extinction probability will increase when Bd is present
	Bd	NA	We did not include covariates on extinction probability of Bd because we did not have a priori hypothesis of what may affect Bd extinction

Abbreviation: Bd, *Batrachochytrium dendrobatidis*.

## 2 Methods

### 2.1 Study Sites

**FIGURE 1:**
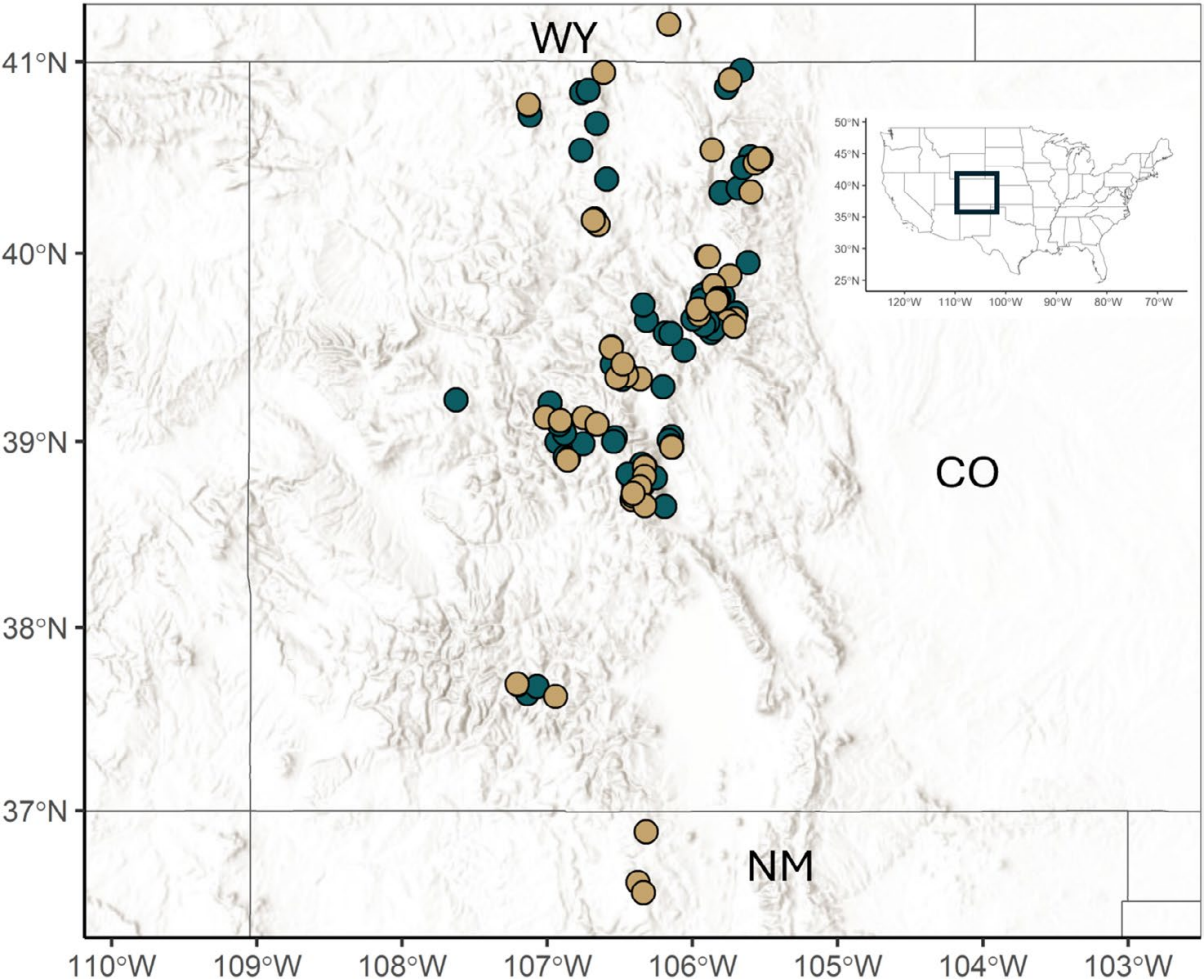
Map of historical boreal toad breeding locations in the southern Rocky Mountain Region, spanning from southern Wyoming (WY) through Colorado (CO) to northern New Mexico (NM) in the United States (inset map). Green points indicate breeding sites with detection/non‐detection data that were included in the model, and light brown points indicate breeding sites without detection data. Map source: Esri world hillshade. Datum: World Geodetic System of 84, Projection: Pseudo‐Mercator.

We worked with the BTCT in an iterative process to delineate spatial boundaries for 161 current and historical boreal toad breeding sites across the SRM (Figure 1). Elevation of sites extended from 2438 to 3731 m. Delineated surface area of sites ranged from 208 to 153,847 m^2^ and sites consisted of wetlands, ponds, lakes, and river or stream channels that ranged from ephemeral to permanent water bodies.

### 2.2 Boreal Toad Breeding and Bd Detection/Non‐Detection Data

The authors acquired all boreal toad and Bd data for analysis from Colorado Parks and Wildlife (Colorado Parks and Wildlife 2024) and did not collect data directly, thus animal care and use approval was not required by our institutions. Following a standard occupancy modeling framework (MacKenzie et al. 2003), we considered each year as a “primary period” between which the occupancy status for boreal toad breeding or Bd at a site can change via local extirpation or local colonization. We treated visual surveys within each year for boreal toads as “secondary occasions” during which the species' occurrence is assumed to be static. The number of visual surveys at each site within a year ranged between 0 and 10, and the average was 2.5 per year. Samples for Bd were taken primarily from swabs of adult or terrestrial stage toads in the system (Mosher, Bailey, Hubbard, and Huyvaert 2018). Thus, to avoid identifiability issues resulting from the confounding detection of toads and Bd described by Mosher, Bailey, Hubbard, and Huyvaert (2018), we used evidence of toad breeding (i.e., egg masses, tadpoles, or young of year observed) as detections of toad occurrence (i.e., if only juveniles older than a year or adult toads were detected, we considered detection to be 0). This approach aligns with previous occupancy modeling efforts for this system (Gerber et al. 2018; Mosher, Bailey, Muths, and Huyvaert 2018), and allowed us to quantify the probability that breeding ceased at a site (i.e., while there may be some adults, breeding is not occurring), a scenario which has occurred at multiple sites within our study system (Crockett 2023).

The granularity of the Bd data was coarse, and often included only the total number of samples and the proportion of positive samples at a site in a given year (i.e., sampling dates were missing). Thus, to develop a detection history for Bd, we considered each sample from a site within a year a “secondary occasion” and we randomly assigned Bd detection (or non‐detection) to a secondary occasion based on the proportion of positive samples for the site in the year sampled (sensu Gerber et al. 2018; Mosher, Bailey, Muths, and Huyvaert 2018). For example, if 25 samples were collected from a site in a year and the Bd prevalence was reported as 0.20, we treated the site as having 25 secondary occasions with Bd detections randomly assigned on 5 of those occasions. Furthermore, because the number of Bd samples varied dramatically among sites (0–30), we combined Bd samples into up to 6 secondary occasions and denoted a Bd detection when at least one sample within the occasion was positive (i.e., up to 5 samples were collapsed into a single occasion, Figure S1).

We used the detection/non‐detection data for breeding toads and Bd, along with several climate covariates (described in Section 2.3) to model the probability that each breeding site was in one of four “states”: (1) breeding toads present but no Bd, (2) breeding toads and Bd present, (3) occupied by Bd only (breeding toads not present), or (4) unoccupied (Bd and breeding toads not present). The four states align with previous occupancy analyses for this system (Converse et al. 2017; Gerber et al. 2018; Mosher, Bailey, Muths, and Huyvaert 2018), and details regarding the formulation of the dynamic occupancy are described below in Section 2.5.

### 2.3 Dynamic Occupancy Model Covariate Data Development

To model species‐specific occupancy dynamics, we derived three covariates that we hypothesized could affect boreal toads (*Active season length, Proportion of May cold events*, and *Proportion of years wetland dried*) and two covariates that could affect Bd (*Bd growing degree days* and *Proportion of years wetland dried*) (Table 1). Boreal toads are relatively long‐lived and require multiple years to reach sexual maturity; therefore, we hypothesized that occupancy dynamics are more related to conditions over multiple years than conditions in a single year. Thus, we developed covariates such that the value in year *t* represents changes in that covariate over the prior 15‐year (*t*−1 to *t*−15) timespan (refer to Table 2 for covariate definitions and context). We chose 15 years based on available time series of data and because it represents approximately three generations of boreal toads (Muths and Nanjappa 2005), which we hypothesized was enough time to capture changes in occupancy dynamics.

**TABLE 2:** Description and methods for deriving occupancy model covariates.

Covariate	Description	Equation	Range of values
Active season length	We defined active season length for a single year as the deviation from average active season length described by the 35‐year (1985–2019) climate norm. To derive this covariate, we calculated the cumulative number of days between the last day of −4.4°C temp in spring and first day of −4.4°C temp in autumn for each year between 1985 and 2019 and took the average for each site, which represents the 35‐year climate norm (Equations 1 and 2). We then subtracted the climate norm for the site from the active season length for a given year (Equation 3). Thus, a negative value represents a shorter active season than average, and a positive value represents a longer growing season than average. We further calculated a rolling average of the 15 years prior to year *t* in our time series to represent long‐term changes in active season length in our model. For example, “active season length” in 2001 is the average deviation from the climate norm from 1985 to 2000, “active season length” in 2002 is the average deviation from the climate norm from 1986 to 2001	(1). ${\mathrm{SL}}_{i,t}=\left({\text{autumn}}_{i,t}-{\text{spring}}_{i,t}\right)$ (2). ${\bar{\mathrm{SL}}}_{i}=\frac{\sum_{t=1985}^{2019}{\mathrm{SL}}_{i,t}}{35}$ (3). $\Delta {\mathrm{SL}}_{i,t}={\mathrm{SL}}_{i,t}-\bar{{\mathrm{SL}}_{i}}$	−45.7 to 53.4 days
May cold events	For each year, we calculated the number of “cold events” during the egg laying period (May). A “cold event” is defined as a day with an average minimum temperature at least 2 Standard Deviations (SD) below the 30‐year average minimum temperature for May (Equation 1). We then calculated the proportion of years with cold events for the 15 years prior to year *t* (Equation 2). For example, “cold events” for 2001 is the proportion of years with cold events in May from 1985 to 2000	(1). ${\text{ColdEvent}}_{i,y,d}={\text{MinTemp}}_{i,y,d}<\left(\mu_{i}^{\mathrm{May}}-2\sigma_{i}^{\mathrm{May}}\right)$ (2). ${\text{PropCold}}_{i,t}=\frac{\sum_{y=t-15}^{t-1}{\text{ColdEvent}}_{i,y}}{15}$	0.067 to 0.93
Bd growing degree days	The number of days in year *t* with a minimum temperature above 17°C and a maximum temperature below 27°C. These values represent the thermal breadth of Bd from the literature (Piotrowski et al. 2004; Berger et al. 2004; Haver et al. 2022)	(1). ${\mathrm{DD}}_{i,t}=\left\{\begin{array}{c} 1\;\text{if}\;T_{\min,i,t,d}>17^{o}C\;\text{and}\;T_{\max,i,t,d}<27^{o}C\\ 0\;\text{otherwise} \end{array}\right.$ (2). $\;{\mathrm{GDD}}_{i,t}=\sum {\mathrm{DD}}_{i,t}$	9 to 143 days
Proportion of years dried	The proportion of the last 15 years that the site was estimated to have dried from the hydrology models. This is a rolling value, such that the value for year *t* is derived from the probabilities of drying between years *t*−1 and *t*−15	(1). ${\mathrm{Dry}}_{i,y}=\left\{\begin{array}{c} 1\;\text{if}\;{\text{JulySA}}_{i,y}=0\\ 0\;\text{Otherwise} \end{array}\right.$ (2). ${\text{PropYrsDry}}_{i,t}=\frac{\sum_{y=t-15}^{t-1}{\mathrm{Dry}}_{i,y}}{15}$	0 to 1

*Note:* μ, 30‐year mean May minimum temperature. JulySA, surface area estimate in July (in m^2^).

Abbreviations: DD, degree day; GDD, growing degree day; SL, season length.

We hypothesized that the *Proportion of years dried* over the prior 15 years could affect initial occupancy (i.e., the probability of a site being occupied by breeding toads in year one of the study) and subsequent extinction probability (i.e., the probability that that toads cease to breed, given the site previously supported breeding) via repeated failed breeding (e.g., ponds drying before metamorphosis occurs). Similarly, we hypothesized that the *Proportion of years with May cold events* over the prior 15 years could cumulatively reduce breeding success if egg masses freeze and die (Muths et al. 2017), also influencing toad extinction probabilities. Further, we hypothesized that increases in *Active season length* (deviations from long‐term norm) may result in a phenological mismatch between available food resources for terrestrial boreal toads, reducing local populations to the point of extirpation. For Bd, we hypothesized that as summation of the number of the days with a maximum temperature above 17°C but below 27°C, Table 2 increases, the probability of initial Bd occupancy and colonization (i.e., the probability that Bd is present at a site, given it was absent the year prior) would increase given that winter temperatures at high elevation sites in the SRM are typically below the thermal optimum of Bd for, likely limiting Bd presence (Berger et al. 2004; Haver et al. 2022; Mosher, Bailey, Muths, and Huyvaert 2018; Piotrowski et al. 2004). Note, we did not average *Bd growing degree days* over the prior 15 years because we hypothesized that for Bd, the number of growing degree days during the current season would likely affect colonization of Bd more than past conditions. We also hypothesized that the *Proportion of years dried* would have a negative effect on Bd initial occupancy and colonization probability because, despite being able to persist in mud and sand (Johnson and Speare 2005), Bd is less likely to persist in environments that dry in consecutive years (Pujol‐Buxó and Montori 2025).

We used gridMET data (Abatzoglou 2013) to derive covariates for *Active season length*, *May cold events*, and *Bd growing degree days* for current climate conditions (refer to Climate Data Extraction, Supporting Information). However, data were not available for *Proportion of years dried* because there were no available models to predict probabilities of drying for each wetland in our dataset. Therefore, we developed a hydroperiod model (refer to section 2.4 below) to estimate the annual probability of drying at individual sites. For all covariates (*Active season length*, *May cold events*, *Bd growing degree days* and *Proportion of years dried*), we derived or calculated historical and current values for all boreal toad breeding sites between 2000 and 2019.

To generate datasets for future predictions, we derived or calculated all covariate values for six General Circulation Models (GCMs) and two Representative Concentration Pathways (RCPs) between 2040 and 2069 (Table S1). We selected six GCMs representing a range of plausible and divergent futures in the SRM region (Clark‐Wolf et al. 2025; Lawrence et al. 2021). Four scenarios were based on previous work by Steen (2017): a hot, wet winter (CanESM2), a warm, wet winter (GFDL‐ESM2M), a warm, dry winter (inmcm4), or a hot, dry winter (IPSL‐CM5A‐LR). We included two additional models that represented plausible changes in spring conditions we hypothesized would affect boreal toad breeding: a hot, dry spring (MIROC‐ESM‐CHEM), or a hot, wet spring (HADGEM2‐ES365). We chose specific GCMs representing the scenarios described above by using the “Future Climate Scatter” web tool (Hegewisch and Abatzoglou 2017) and visualizing changes in temperature and precipitation for each model under RCPs 4.5 (representing a low emissions scenario) and 8.5 (representing a potential upper bound for a high emissions scenario) (Table S1). Acknowledging the uncertainty in future predictions, these divergent climate scenarios and RCP pathways allow us to quantify and compare the magnitude of change in breeding toad and Bd occupancy across a range of more probable to less probable conditions that may help managers in the event of “surprise” trajectories (Lawrence et al. 2021).

### 2.4 Hydroperiod Modeling

We developed a hydroperiod model to predict the annual probability of drying at breeding sites. We assessed the effect of variables including soil moisture, temperature, and aspects of topography (Table S2, refer to climate data extraction methods in Supporting Information) on the probability of drying, using surface area estimates from remotely sensed data (sensu Halabisky et al. 2016) for our known breeding sites as the response variable. The remotely sensed data consisted of bi‐weekly surface water area estimates from May through October between 1985 and 2019 for each site (Kissel, Muths, et al. 2025). We used a Bayesian modeling framework implemented in the package *brms* (Bürkner 2017) for program R v4.3.2 (R Core Team 2024) to create hurdle models that estimate the probability of a wetland drying (logistic model with a logit link) and the total surface water area of the wetland (gamma distribution) in July of a given year (a critical month for boreal toad larval development). We applied a threshold to classify the breeding site as dry (≤ 15% of maximum surface area of the wetland) or not dry (> 15% of maximum surface area of the wetland, Kissel et al. 2020). Four outlier sites were removed from the analysis as they were large managed reservoirs and unlikely to represent natural hydroregimes.

We used a similar parameterization for the logistic and gamma sub‐models within the hurdle model. We eliminated several variables with high (> |0.70|) Pearson correlations (retaining the most ecologically relevant variable, Zuur et al. 2009), resulting in the following variables for modeling: *wetland size, monthly precipitation, monthly mean vapor pressure deficit (vpd), soil water storage, snow water equivalent in the previous winter, snow water equivalent in the previous month, watershed area, area of forested and shrub wetland within the watershed, area of emergent wetland within the watershed, terrain ruggedness index, aspect*, and *continuous heat‐insolation load index (CHILI)* (Table S2). We used two random effects for each sub‐model: a *year* effect and *site* effect. The former accounts for latent variation in climate conditions within each year, while the latter accounts for latent variation in conditions at each site across all years.

We tested several models containing parameters grouped by climate, water balance, topography, CHILI, and wetland, along with several models with combinations of variables drawing from different groups (refer to Climate Data Extraction methods, Table S1). We evaluated model performance using conditional and marginal leave‐one‐out (LOO) adjusted *R* ^2^ with compatibility interval (function *r2_loo* in package *performance*, Lüdecke et al. 2021). We used the best performing model to predict the annual probability of drying and the area of surface water for the month of July for each breeding site between 1985 and 2022. We then used the model to forecast the probability of drying and surface water area for the future time horizon (2040–2069) under the six GCMs (Table S1) and two RCPs (4.5 and 8.5).

To assess potential hydroperiod shifts under future conditions, we classified breeding sites as ephemeral, intermediate, perennial, and permanent based on their likelihood to shrink in the future relative to the current area. For each year (*t*) and breeding site (*i*) we used the equation:

$$\text{Area Reduction}\;{\left(\mathrm{AR}\right)}_{t,i}=\frac{1-\left({\text{maximum area}}_{i}-\text{July}\;{\text{predicted area}}_{t}\right)}{{\text{maximum area}}_{i}}$$

where maximum area is the originally delineated area of the breeding site (Kissel, Muths, et al. 2025). We then calculated the mean AR for each breeding site and climate scenario and classified ponds as ephemeral (*AR* ≤ 0.03), intermediate (0.03 < *AR* ≤ 0.33), perennial (0.33 < AR ≤ 0.70), and permanent (*AR* ≥ 0.7) (Lee et al. 2015).

### 2.5 Dynamic Occupancy Model

We fitted a Bayesian multi‐state dynamic co‐occurrence occupancy model following the framework developed by Fidino et al. (2019). We used detection/non‐detection data for boreal toad breeding and Bd, and corresponding covariates between 2001 and 2019, from 75 sites across the breeding range of the species. The model estimated the probability that each site is in one of four “states”: (1) breeding boreal toads present, (2) Bd and breeding boreal toads present, (3) occupied by Bd only (breeding toads not present), or (4) unoccupied (Bd and breeding toads not present). This model quantifies the direct effects of Bd by modeling the influence of the presence of Bd on boreal toad breeding dynamics (extinction and colonization) while accounting for imperfect detection in both species (toads and Bd). For example, a transition of a site from state two (breeding toads and Bd) to state three (Bd only) is the extinction probability for toads (estimated as a function of climate covariates) multiplied by a separate term derived from the probability that the presence of Bd affects extinction of breeding toads. For additional details, refer to Fidino et al. (2019) for model derivation and Gerber et al. (2018) for state definitions for this system. The initial occupancy probability (i.e., occupancy in 2001) was estimated directly, and in subsequent years extinction and colonization probabilities for both species (toad breeding, Bd) were estimated. The occupancy state of the site in years two through 19 was derived via estimated state‐specific colonization and extinction probabilities. We modified the Fidino et al. (2019) model in three ways: (1) we included time‐varying covariates, (2) we used an informed prior for Bd extinction probability because the probability was expected to be low due to the ability of Bd to persist in the environment (Johnson and Speare 2005), and (3) we did not model the effect of boreal toad presence on colonization and extinction rates of Bd because we did not have a priori hypotheses on how the presence of boreal toads would affect colonization or extinction probably of Bd, and Bd data were sparse. We explored the effects of the covariates described above on the probability of initial occupancy for both breeding toads and Bd, as well as the probability of colonization and extinction for breeding toads and Bd (Table 1).

We predicted the site‐specific occupancy states for 152 historical and current breeding sites (out of the original 161 sites) because covariate data were not available for nine sites. We calculated occupancy state predictions for the current time period (2001–2019) as well as for the future time period (2055–2069), using the climate models described above. For the 75 sites with detection/non‐detection data used to fit the model, we made predictions using the site‐specific value for the intercept estimated by the model and site‐specific covariates. For the remaining 77 sites that were not included in the model (i.e., without detection/non‐detection data), we used site‐specific covariates and the mean estimated intercept from the model (i.e., not site‐specific) to make predictions. Thus, the predictions for the 75 sites included in the model contain more site‐specific information than the 77 sites not included in the model. We further summarized occupancy results by “mountain range,” a regional management unit defined by the BTCT that includes mountain ranges in the SRM (but also the Grand Mesa in Colorado, a geographical feature that is not defined as a mountain range, Crockett 2023). A stated goal of the BTCT is to maintain at least one occupied breeding site in each mountain range and the Grand Mesa (Crockett 2023). Our ability to summarize occupancy results at this level now and under future climate conditions allowed us to explore regional areas of interest that may be more at risk of losing toad breeding sites.

## 3 Results

### 3.1 Hydroperiod Modeling

The best performing hydroperiod model based on LOO was the global model, which included variables across all groups (Table S3). The global model LOO‐adjusted conditional *R* ^2^ (accounting for both fixed and random effects) was 0.929 (95% credible interval [CI]: 0.910–0.946), while the fixed effects had a LOO‐adjusted marginal *R* ^2^ of 0.440 (95% CI: 0.299–0.544). Thus, more than half of the predictive power in the model was due to the random variables *year* and *site*. The random effect of *year* explained less variance in the data compared to the random effect of site (residual variance for *year* = 0.514 and for *site* = 0.865).

The probability of pond drying in July was greatest for smaller ponds (small wetland area, Figure S2) located in watersheds with a higher area of emergent wetland vegetation and a low terrain ruggedness index; ponds were less likely to dry in July during years with greater snowfall during the preceding winter and the month of June, particularly when vapor pressure deficit was low during July (Figure S1, Table S3). Surface area was positively associated with wetland area, but the estimated coefficients of all other variables had credible intervals that overlapped zero, suggesting weaker effects (Table S3).

**FIGURE 2:**
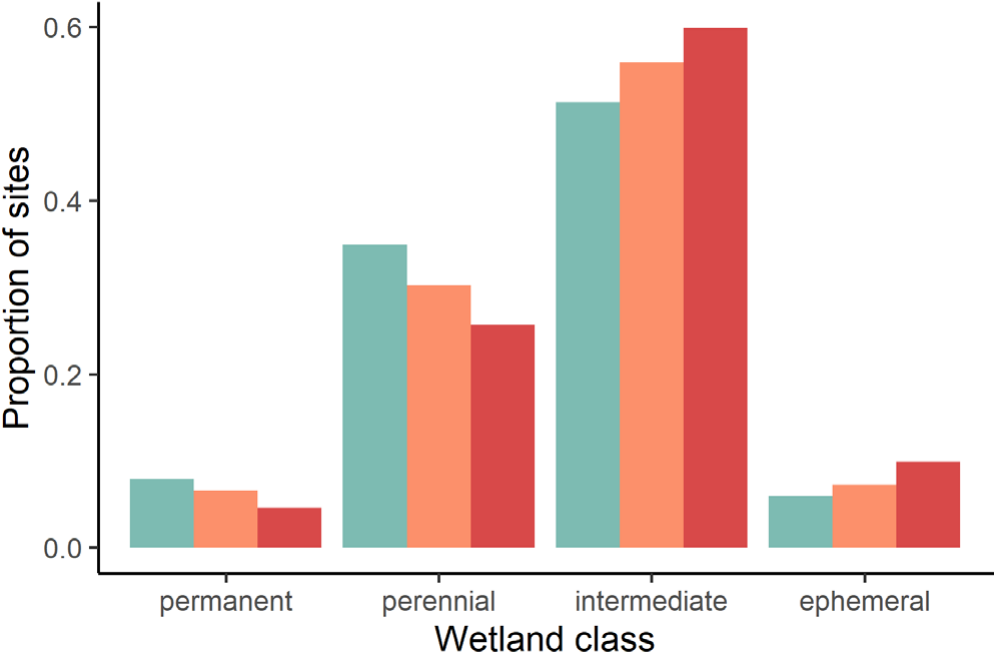
Shift in wetland hydroperiod class for boreal toad breeding sites under historical (1985–2022, green bars) and (2040–2069) climate scenarios (Representative Concentration Pathway 4.5 [orange bars] and 8.5 [red bars]).

We found that the probability of drying increased for the 2040–2069 horizon relative to the current baseline, with higher drying under the RCP 8.5 compared to RCP 4.5 scenarios (Figure S3). Under the RCP 8.5 scenario, the proportion of *ephemeral* ponds almost doubled, from 0.06 to 0.10, *intermediat*e ponds increased slightly from 0.51 to 0.60, while the proportion of *perennial* and *permanent* ponds decreased from 0.35 to 0.25 and 0.08 to 0.05, respectively. The RCP 4.5 scenario showed similar patterns, but the results were more moderate compared to RCP 8.5 (Figure 2).

### 3.2 Dynamic Occupancy Modeling

#### 3.2.1 Initial Occupancy—Boreal Toad Breeding

The posterior distribution of coefficient estimates for the linear term of *proportion of years dried* on the probability of initial occupancy of breeding boreal toads suggested a weakly negative relationship (indicated by 95% CI that overlap zero), but the posterior distribution of coefficient estimates for the quadratic term suggested a weakly positive relationship (Figure S4). Contrary to our hypothesis, initial breeding toad occupancy (in 2001) was highest in permanent wetlands that never dried, and perhaps in strictly ephemeral wetlands (though this estimate is imprecise; Figure S4). Occupancy probabilities were slightly lower for wetlands with intermediate or perennial hydroperiods (i.e., those with intermittent drying).

#### 3.2.2 Probability of Colonization—Boreal Toad Breeding

The posterior distribution of coefficient estimates for the linear term *proportion of years dried* on the probability of colonization of breeding toads was strongly positive, but the posterior distribution of coefficient estimates for the quadratic term was strongly negative (Figure S5). Colonization probabilities were lower at ephemeral sites (i.e., sites that dried the most frequently) and permanent sites that never dried, and highest at sites that dried ~40% of the time in the last 15 years (Figure S5). However, uncertainty in colonization probability estimates increased as the proportion of years dried increased.

#### 3.2.3 Probability of Extinction—Boreal Toads

The posterior distribution of coefficient estimates for the linear term of *proportion of years dried* on the probability of extinction of breeding toads was strongly positive, but the posterior distribution of coefficient estimates for the quadratic term was strongly negative (Figure S6). Contrary to our hypothesis, extinction probability was highest at sites that dried ~47% of the time in the last 15 years, and similar to colonization, extinction probability was lower in sites that dried more frequently or were more permanent (although uncertainty was higher for more permanent ponds, Figure S6). Also contrary to our hypothesis, the posterior distribution of the coefficient estimates for the effect of the number of *May freeze events* was weakly negative (Figure S6), and extinction probability was highest (mean: 0.52, 95% CI 0.23–0.80) when the proportion of years with freeze events was near zero. The posterior distribution of the coefficient estimates for the effect of *season length* was strongly positive (Figure S6), indicating that as season length increases, the probability of extinction increases. Our model indicates extinction probability is 0.82 (95% CI 0.68–0.92) for a *season length* that is 15 days greater than the 35‐year average season length. Conversely, the extinction probability is 0.10 (95% CI 0.04–0.20) for a season length that is 15 days less than the 35‐year average. Finally, the posterior distribution of the effect of Bd presence on breeding toad extinction probability was weakly positive (mean probability = 0.12, 95% CI: −2.66 to 2.5).

#### 3.2.4 Initial Occupancy—Bd

The posterior distribution of the coefficient estimates for *growing degree days* and *proportion of years dried* with respect to the probability of initial occupancy of Bd were both weakly positive (Figure S7). The probability of initial occupancy of Bd ranged from a mean of 0.34 (95% CI: 0.22–0.50) when the proportion of years dried was zero, to a mean of 0.52 (95% CI: 0.14–0.89) when the proportion of years dried was 0.98. The probability of initial occupancy of Bd ranged from 0.04 (95% CI: 0.01–0.11) when the average number of *growing degree days* in the last 15 years was 9.42, to 0.10 (95% CI: 0.03–0.24) when the average number of *growing degree days* in the last 15 years was 137.2.

#### 3.2.5 Probability of Colonization—Bd

Similar to initial occupancy probability, the posterior distribution of the coefficient estimates for *growing degree days* on the probability of colonization of Bd was weakly positive (Figure S8). However, the posterior distribution of the coefficient estimates for the *proportion of years dried* on the probability of colonization of Bd was centered on zero, suggesting no effect.

#### 3.2.6 Current and Future Occupancy States

**FIGURE 3:**
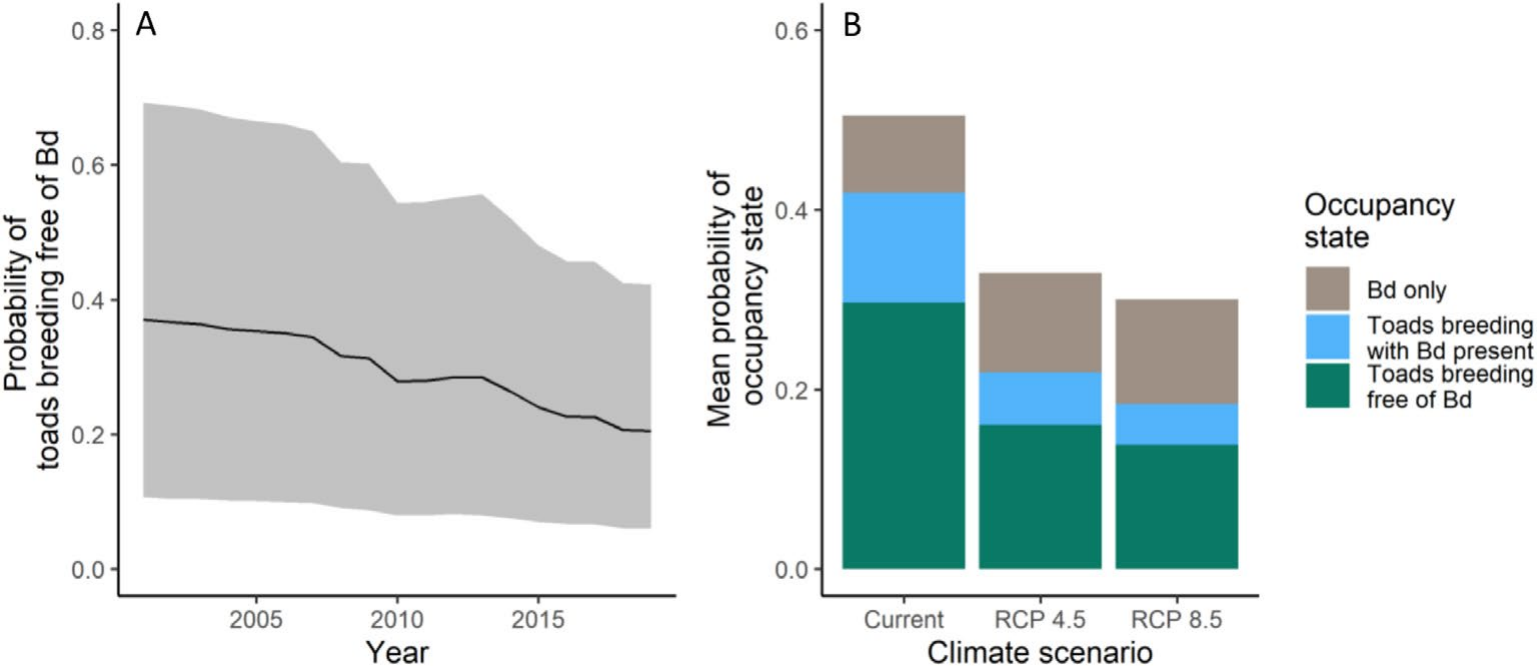
Probability of a historical boreal toad breeding site within the southern Rocky Mountain region (SRM) being occupied by breeding toads free of Bd between 2001 and 2019 (gray shaded region represents 95% credible interval, panel A). Comparison of occupancy state probabilities between current and Representative Concentration Pathways (RCP) 4.5 and RCP 8.5 (2055–2069) (panel B). Green bars = “breeding toads free of Bd,” blue bars = “toads with Bd present,” gray bars = “Bd alone.”

The mean probability that a site within the SRM is occupied by breeding toads alone (state 1) declined from 0.37 (95% CI: 0.11–0.69) in 2001 to 0.21 (95% CI: 0.06–0.43) in 2019, a decline of 43.24% (Figure 3a). The mean probability that a site within the SRM was occupied by breeding toads and Bd (state 2) declined from 0.15 (95% CI: 0.015–0.38) in 2001 to 0.08 (95% CI: 0.01–0.25) in 2019, concurrent to the decline in breeding toad occupancy.

The overall mean occupancy probability (Figure 3b) of a site in the SRM being occupied by breeding toads alone (state 1) under current climate conditions (i.e., 2001–2019) was 0.30 (95% CI: 0.08–0.57). We used the ensemble mean of the 6 climate models (Table S1) to predict future occupancy (i.e., 2055–2069), and we found that the overall mean probability of breeding toad occupancy declined to 0.16 (95% CI: 0.04–0.34) under the RCP 4.5 climate scenario and 0.14 (95% CI: 0.04–0.30) under the RCP 8.5 climate scenario (Figure 3b).

**FIGURE 4:**
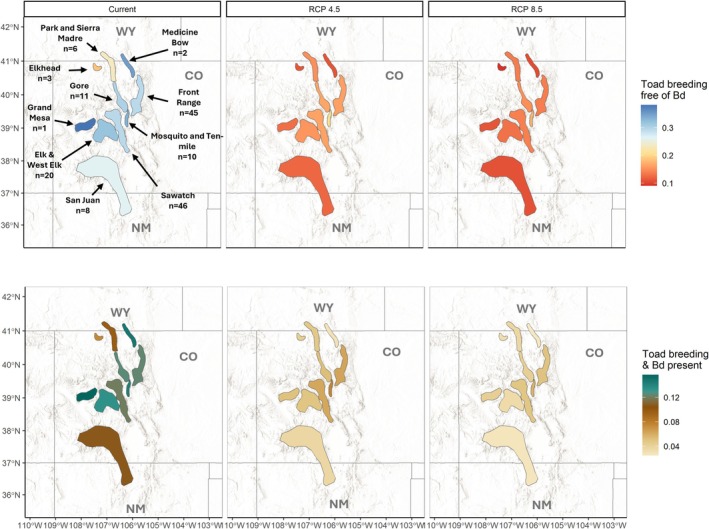
Maps indicating the mean probability of a site within a mountain range being in one of two states (occupied by breeding toads free of Bd, occupied by breeding toads and Bd present) under current climate conditions (2001–2019) and two future climate scenarios between 2055 and 2069: Representative Concentration Pathway (RCP) 4.5 (lower emissions) or 8.5 (higher emissions). The names of the mountain ranges and the number of sites within each mountain range are depicted in the top left panel for reference. Map source: Esri world hillshade. Datum: World Geodetic System of 84, Projection: Pseudo‐Mercator.

The mean probability of a site being occupied by breeding toads only (state 1) within a mountain range under current conditions ranged from 0.19 (95% CI: 0.02–0.49) for the Elkhead Mountains (*n* = 3 sites) to 0.38 (95% CI: 0.11–0.66) for the Grand Mesa (*n* = 1 site, Figure 4, Table S4). The mean probability of a site being occupied by breeding toads and Bd (state 2) ranged from 0.07 (95% CI: 0.004–0.28) for the Elkhead Mountains to 0.16 (95% CI: 0.02–0.37, Figure 4) for the Grand Mesa. The Sawatch Range and the Front Range contain over half of the 152 breeding sites for which we made predictions (*n* = 46 and *n* = 45 respectively). For the Sawatch Range, the mean probability of a site being occupied by breeding toads only (state 1) declined by 49% between 2001 and 2019. The overall mean probability of a site being occupied by breeding toads only (state 1) in the Sawatch Range under current conditions was 0.30 (95% CI: 0.08–0.58; Figure 4). Under future climate conditions using the ensemble mean of 6 GCMs (Table S1), the mean probability that breeding toads alone (state 1) occupied a site within the Sawatch Range was 0.16 (95% CI: 0.03–0.38) under the RCP 4.5 scenario, or 0.14 (95% CI: 0.03–0.33) under the RCP 8.5 scenario (Figure 4). For the Front Range, the mean probability that breeding toads alone (state 1) occupied a site within that mountain range declined by 40% between 2001 and 2019. The overall mean probability that breeding toads alone occupied a site within the Front Range was 0.29 (95% CI: 0.08–0.57). Under future climate conditions, the mean probability that breeding toads alone occupied a site within the Front Range was 0.16 (95% CI: 0.04–0.36) under the RCP 4.5 scenario, or 0.14 (95% CI: 0.03–0.31) under the RCP 8.5 scenario (Figure 4).

The biggest decline in the probability of occupancy for state 1 (breeding toads only) under future climate change was in the Grand Mesa, from a mean of 0.38 (95% CI: 0.11–0.66) under current conditions to a mean of 0.13 (95% CI: 0.02–0.38) under the RCP 4.5 scenario and mean of 0.11 (95% CI: 0.01–0.33) under the RCP8.5 scenario; however the Grand Mesa only consists of a single site (Table S4). The second biggest decline in breeding toad occupancy (state 1) under future climate change was the Medicine Bow Mountains (*n* = 3 sites), which declined from a mean of 0.35 (95% CI: 0.14–0.55) under current conditions to 0.11 (95% CI: 0.06–0.18) under the RCP 4.5 climate scenario. Similarly, occupancy declined to 0.11 under the RCP 8.5 scenario, but the uncertainty around the estimate was slightly lower (95% CI: 0.06–0.16). The Elkhead Mountains had the lowest decline in occupancy under future conditions, declining from a mean of 0.19 (95% CI: 0.02–0.49) under current conditions to 0.11 (95% CI: 0.01–0.33) under the RCP 4.5 scenario or 0.09 (95% CI: 0.01–0.27) under the RCP 8.5 scenario (Table S4).

## 4 Discussion

Our analysis indicates a > 43% decline in the probability of occupancy for breeding boreal toads at a given site across the SRM between 2001 and 2019, which aligns with empirical observations over the last two decades (Crockett 2023). While this decline has largely been attributed to Bd (Hardy et al. 2023; Muths et al. 2003; Scherer et al. 2005), we highlight the strong effect of increasing active season length on boreal toad extinction probability (Figure S6). This result may appear counterintuitive because a longer active season suggests greater access to resources (i.e., food) and/or time to find suitable hibernacula before winter. However, for high elevation amphibians like boreal toads, this result could indicate a phenological mismatch, when food resources are limited in the fall, but boreal toads are still active due to high temperatures (Church et al. 2007; Reading 2007; Williams et al. 2015). Extended monitoring and studies designed to quantify changes in available food resources at the beginning and end of the active season could help validate this for toads and other montane amphibian species. In the SRM, boreal toads occur at high elevations in a snow‐dominated environment, with relatively little precipitation during the active season (Mote et al. 2005). This may result in a drier landscape as the active season length is extended into the fall, increasing desiccation risk, particularly for juvenile toads (Lertzman‐Lepofsky et al. 2020; Greenberg and Palen 2021).

We found a weakly negative relationship between the proportion of years with freezing events in May and breeding toad extinction probability, suggesting that the probability of extinction declines as the proportion of years with freeze events in May increases. While contrary to our hypothesis, the pattern is consistent with our findings for active season length. These covariates were not highly correlated and both were included in the model. However, as temperatures warm and the active season length increases, the number of freeze events is likely to decrease. Thus, while we hypothesized that May freeze events could increase mortality at the egg stage, our results indicate that the demographic effect of egg mortality is unlikely to be greater than the effect of warmer and longer active seasons on the probability of breeding toad extinction.

Our modeling results suggest that the probability of initial occupancy is lowest for sites that dry ~50% of the time (within the prior 15 years), and that the probability of colonization and extinction of breeding boreal toads is highest for these perennial and intermediate wetlands (Figures S5 and S6) even though they have historically supported the majority of boreal toad breeding habitat (Figure 2). Under future climate scenarios, our hydroperiod models predict that sites may shift to wetland classes that dry more frequently (e.g., permanent to perennial, perennial to intermediate, Figure 2), which is likely to exacerbate declines in breeding toad occupancy if current relationships between occupancy dynamics and wetland drying hold in the future.

The effect of Bd on the extinction probability of breeding toads was weaker compared to previous analyses (Gerber et al. 2018; Mosher, Bailey, Muths, and Huyvaert 2018). However, prior analyses did not include climate covariates, which may be explaining some of the variation previously attributed to (or confounded with) Bd. For example, as season length increases, Bd is also more likely to grow, reproduce, and be transmitted among co‐occurring or interacting amphibians, particularly at higher elevation sites where season length and temperatures may have previously limited Bd growth (Mosher, Bailey, Muths, and Huyvaert 2018). Additionally, our updated analysis leveraged 10 more years of data, and the longer time series may have allowed us to better capture occupancy dynamics. However, similar to previous analyses, Bd data were coarse (i.e., did not include sampling dates) and largely tied to swabbing adult toads. Bd sampling at sites independently of toads, and more detailed data recording could lead to a better understanding of Bd occupancy dynamics in the SRM, and allow researchers to tease apart potential effects of seasonality on Bd detection.

Neither the proportion of years dried nor the number of Bd growing degree days had a strong effect on initial occupancy or colonization of Bd. This may reflect the sparseness of the Bd data or indicate that the covariates are not good predictors of Bd occupancy dynamics. Given the complexity of the Bd life cycle, including introduction to the system, infection rate of hosts, and pathogen growth within hosts (e.g., ability of hosts to mediate growth), course climate metrics that can be derived at the temporal scale that align with occupancy data may be unable to capture the finer scale abiotic and biotic conditions driving the presence and persistence of Bd (Gajewski et al. 2021).

We predict that declines in occupancy in the SRM are likely to continue under future climate change. While the mean probability of occupancy declined by > 43% between 2000 and 2019 in the SRM, we predict an additional decline of ~47% (from 0.30 to 0.16) under the RCP 4.5 scenario or an additional ~53% (from 0.30 to 0.14) under the RCP 8.5 scenario. Notably, our predictions for the more extreme RCP 8.5 scenario are only 6% higher than predictions for the RCP 4.5 scenario, indicating that even moderate increases in warming and drying could be consequential. The northwest and southernmost mountain ranges had the lowest occupancy probabilities under current conditions, while the mountain ranges in the central part of the SRM tended to have higher occupancy probabilities for both breeding toads only and breeding toads + Bd (Figure 4). However, occupancy in all mountain ranges is predicted to decline under future climate scenarios (Figure 4). Further, the more central mountain ranges that contain the bulk of boreal toad breeding sites (the Front Range in particular) are located in areas with high anthropogenic disturbance (e.g., major highways and recreational opportunities) that could have additive or synergistic effects with both Bd (i.e., higher probability of disease transfer) and climate (e.g., higher risk of burning, Jaffe et al. 2024; Lacey et al. 2025).

The declines observed between 2001 and 2019, coupled with the predictions of further declines in the future due to the combined effect of changing hydroperiod, increasing active season length, and Bd prevalence, indicate increased uncertainty in the persistence of breeding boreal toads in the SRM. However, the BTCT has developed a decision support tool that quantifies the probability of success of several conservation actions and is robust to highly uncertain Bd dynamics (Gerber et al. 2018, 2024). We build on this decision support tool by elucidating spatiotemporal trends in occupancy patterns with respect to climate covariates. The output from our analyses is accessible to the BTCT via a web tool developed with input from boreal toad managers (Lacey et al. 2025). The tool provides (1) background information on our modeling approaches, (2) geographic context for the analysis in the form of an interactive map visualizing boreal toad breeding and translocation sites, watershed drying patterns and watershed burn probabilities (Lacey et al. 2025), and (3) summary statistics and time series plots of current and future estimates of boreal toad and Bd occupancy at different management levels (individual site, mountain range, and all sites combined).

In this analysis, we demonstrate the utility of wetland‐specific remote sensing data (Kissel, Muths, et al. 2025), coupled with large, publicly available long‐term climate and climate‐derived datasets (Abatzoglou 2013; Abatzoglou and Brown 2012; Alder and Hostetler 2021) to develop predictive models of hydroperiods in the SRM and to quantify hydroperiod shift under several GCMs and RCPs for future time periods. The remote sensing analyses (Kissel, Muths, et al. 2025), the long‐term daily historical gridMET data (Abatzoglou 2013), and future climate data derived via multivariate adaptive constructed analog (Abatzoglou and Brown 2012; Alder and Hostetler 2021), provided the opportunity for fine‐scale temporal and spatial predictions of surface water area and the probability of drying under recent past conditions, current conditions, and future forecasts. The hydroperiod predictions were driven largely by pond size and the amount of snowpack the previous winter, which was expected given that ponds in this alpine system are fed largely via snowmelt. Another important driver of pond drying was the vapor pressure deficit, which is a measure of how much moisture the air can hold compared to how much is actually in the air. This moisture‐related metric is temperature‐dependent, so that at a similar air humidity value, the vapor pressure deficit is greater under warmer temperatures, thus leading to greater potential for evaporation.

Overall, the results of the hydroperiod models are intuitive as they suggest that smaller ponds are at greater risk of drying when snowpack during the previous winter is lower and July temperatures are higher, contributing to higher water pressure deficit and increased evaporation. The hydroperiod predictions for the 2040–2069 horizon align with our hypothesis that the likelihood of drying increases, particularly under the higher emissions RCP 8.5 scenario. While there was high variability in hydroperiod predictions between GCMs, the proportion of ponds switching to less permanent and more ephemeral increases overall (Figure 2).

Quantifying the magnitude of the effects of climate change on species or ecosystems is challenging given the uncertainties and complexities of the environment, particularly in montane regions (Wolkovich et al. 2014), yet developing relevant scenarios can be useful and informative for making management decisions (Clark‐Wolf et al. 2025). Ensuring that models align with the temporal and spatial scales at which changes could be expected given species' life history traits (e.g., responses may not be observed after a single generation, or at a small number of sites) can yield stronger inference (Albaladejo‐Robles et al. 2023; Buderman et al. 2023; Jackson et al. 2022). Additionally, focusing on a targeted number of divergent GCMs as opposed to ensemble means of all possible GCMs (*n* = 40) may result in predictions that are more robust to “surprise” trajectories (Lawrence et al. 2021).

This study combined long‐term, empirical data collected by a collaborative team (BTCT) with novel remotely sensed data to produce a decision support tool for managing an imperiled amphibian species now and in the future. Our framework demonstrates the importance of long‐term monitoring by multi‐agency collaborations, particularly of long‐lived species, to generate critical data for making informed decisions. While the boreal toad and Bd data curated by the BTCT have contributed to several other informative analyses (Cayuela et al. 2021; Converse et al. 2017; Crockett et al. 2020; Gerber et al. 2024, 2018; Hardy et al. 2023; Mosher, Bailey, Muths, and Huyvaert 2018; Muths et al. 2017), this analysis is the first to elucidate the demographic effects of climate change and explore spatial patterns in a hierarchical manner (i.e., individual sites, mountain ranges, SRM). For example, our analysis supports the goal of the BTCT to maintain at least one occupied breeding site in each mountain range across the SRM (Crockett 2023) by allowing managers to look at trends and future predictions across mountain ranges to guide decisions targeting this goal. The supporting web tool allows managers from different jurisdictions to focus on site‐specific estimates and future predictions to inform management of individual sites, while information at the levels of a “mountain range” and the SRM can help inform broader conservation efforts.

## Author Contributions

**Amanda M. Kissel:** conceptualization (equal), data curation (equal), formal analysis (lead), funding acquisition (lead), methodology (lead), visualization (lead), writing – original draft (lead), writing – review and editing (lead). **L. Mae Lacey:** data curation (equal), formal analysis (equal), methodology (supporting), software (lead), visualization (supporting), writing – original draft (supporting), writing – review and editing (supporting). **Viorel D. Popescu:** data curation (equal), formal analysis (equal), methodology (supporting), visualization (supporting), writing – original draft (supporting), writing – review and editing (supporting). **Marissa A. Dyck:** data curation (equal), formal analysis (equal), methodology (supporting), visualization (supporting), writing – original draft (supporting), writing – review and editing (supporting). **Larissa L. Bailey:** conceptualization (equal), data curation (equal), formal analysis (supporting), methodology (supporting), writing – review and editing (supporting). **Erin Muths:** conceptualization (equal), funding acquisition (equal), methodology (supporting), project administration (lead), supervision (lead), writing – original draft (supporting), writing – review and editing (supporting).

## Funding

This work was supported by the North Central Climate Adaptation Science Center.

## Conflicts of Interest

The authors declare no conflicts of interest.

## Supporting information


**Figure S1:** Schematic diagram illustrating the process for randomly assigning *Batrachochytrium dendrobatidis* (Bd) detection based on the total number of samples and total number of positives reported for each site. Randomization was necessary because dates of sampling were not reported. We further collapsed the capture histories by combining five occasions/samples into a single occasion to avoid computationally expensive model runs.
**Figure S2:** Marginal effects plots of coefficients included in the top hydroperiod model for the probability of drying.
**Figure S3:** Change in probability of pond drying from historical to 2040–2069 under six Global Circulation Models (GCM) and two Representative Circulation Pathways (RCP): (a) RCP4.5 and (b) RCP8.5
**Figure S4:** Plot of the posterior coefficient estimates for the effect of the quadratic term of the proportion of years dried on the probability of initial occupancy of breeding toads at a site (a) and associated marginal effects plot (b). In panel a, the thick blue line represents the median coefficient estimate and the shaded blue areas represent the 95% credible interval. In panel b, the solid black line represents the mean probability of occupancy for a range of values for the covariate, and the shaded gray area represents the 95% credible interval.
**Figure S5:** Plot of the posterior coefficient estimates for the effect of the quadratic term of the proportion of years dried on the probability of colonization of breeding toads at a site (a) and associated marginal effects plot (b). In panel a, the thick blue line represents the median coefficient estimate and the shaded blue areas represent the 95% credible interval. In panel b, the solid black line represents the mean probability of occupancy for a range of values for the covariate, and the shaded gray area represents the 95% credible interval.
**Figure S6:**. Plot of the posterior coefficient estimates for the effects of each covariate on the probability of extinction of breeding toads at a site (a) and associated marginal effects plot (b–d). In panel a, the thick blue line represents the median coefficient estimate and the shaded blue areas represent the 95% credible interval. In panels b–d, the solid black line represents the mean probability of occupancy for a range of values for the covariate, and the shaded gray area represents the 95% credible interval.
**Figure S7:** Plot of the posterior coefficient estimates for the effects of each covariate on the probability of initial occupancy of *Batrachochytrium dendrobatidis* (Bd) at a site (a) and associated marginal effects plot (b, c). In panel a, the thick blue line represents the median coefficient estimate and the shaded blue areas represent the 95% credible interval. In panels b and c, the solid black line represents the mean probability of occupancy for a range of values for the covariate, and the shaded gray area represents the 95% credible interval.
**Figure S8:** Plot of the posterior coefficient estimates for the effects of each covariate on the probability of colonization of *Batrachochytrium dendrobatidis* (Bd) at a site (a) and associated marginal effects plot (b, c). In panel a, the thick blue line represents the median coefficient estimate and the shaded blue areas represent the 95% credible interval. In panels b and c, the solid black line represents the mean probability of occupancy for a range of values for the covariate, and the shaded gray area represents the 95% credible interval.
**Table S1:** Description of the six General Circulation Models representing different plausible future climate scenarios in the southern Rocky Mountains (Hegewisch and Abatzoglou 2017).
**Table S2:** Names and descriptions of variables used for hydroperiod modeling.
**Table S3:** Coefficient estimates for the top hurdle model predicting hydroperiod (probability of drying) and area of standing water. Std. Error, standard error.
**Table S4:** Comparison of mean occupancy values for each state under current and future climate scenarios for each mountain range containing boreal toad breeding sites in the southern Rocky Mountains. “Bd only” refers to the state in which *Batrachochytrium dendrobatidis* is present but breeding toads are not. RCP, Representative Concentration Pathway.

## Data Availability

The occupancy data cannot be provided publicly because boreal toads are designated as a Species of Greatest Conservation Need in Colorado, Wyoming, and New Mexico, and data are managed by Colorado Parks and Wildlife. All climate data are publicly available and cited within the manuscript and Supporting Information (Abatzoglou 2013; Abatzoglou and Brown 2012; Alder and Hostetler 2021; Earth Science Data Systems 2025; Hegewisch and Abatzoglou 2017; Theobald et al. 2015; U.S. Fish and Wildlife Service 2021). Model outputs are publicly available at: https://doi.org/10.5066/P1VF3ZX4.
