# Supplementary material for: Disease, Drought, and Warming: A Triple Threat to a Declining High‐Elevation Amphibian
Source: Ecol Evol. 2026 Jun 4;16(6):e73767. doi: 10.1002/ece3.73767 (PMC13238775; doi:10.1002/ece3.73767)
Supplement: Supplementary file 1 — Figure S1: Schematic diagram illustrating the process for randomly assigning Batrachochytrium dendrobatidis (Bd) detection based on the total number of samples and total number of positives reported for each site. Randomization was necessary because dates of sampling were not reported. We further collapsed the capture histories by combining five occasions/samples into a single occasion to avoid computationally expensive model runs. Figure S2: Marginal effects plots of coefficients included in the top hydroperiod model for the probability of drying. Figure S3: Change in probability of pond drying from historical to 2040–2069 under six Global Circulation Models (GCM) and two Representative Circulation Pathways (RCP): (a) RCP4.5 and (b) RCP8.5 Figure S4: Plot of the posterior coefficient estimates for the effect of the quadratic term of the proportion of years dried on the probability of initial occupancy of breeding toads at a site (a) and associated marginal effects plot (b). In panel a, the thick blue line represents the median coefficient estimate and the shaded blue areas represent the 95% credible interval. In panel b, the solid black line represents the mean probability of occupancy for a range of values for the covariate, and the shaded gray area represents the 95% credible interval. Figure S5: Plot of the posterior coefficient estimates for the effect of the quadratic term of the proportion of years dried on the probability of colonization of breeding toads at a site (a) and associated marginal effects plot (b). In panel a, the thick blue line represents the median coefficient estimate and the shaded blue areas represent the 95% credible interval. In panel b, the solid black line represents the mean probability of occupancy for a range of values for the covariate, and the shaded gray area represents the 95% credible interval. Figure S6:. Plot of the posterior coefficient estimates for the effects of each covariate on the probability of extinction of breeding t [file ECE3-16-e73767-s001.docx]

**Disease, drought, and warming: a triple threat to a declining high-elevation amphibian**

Amanda M. Kissel^1*^, L. Mae Lacey^2^, Viorel D. Popescu^3^, Marissa A. Dyck^4^, Larissa L. Bailey^5^, Erin Muths^1^

^1^U.S. Geological Survey, Fort Collins Science Center, 2150 Centre Ave. Bldg C, Fort Collins, CO 80526, USA. Email: akissel@usgs.gov, muthse@usgs.gov

^2^ Conservation Science Partners, 1050 Pioneer Trail, Suite 202, Truckee, CA 96161, USA. Email: mae@csp-inc.org

^3^ School of Professional Studies and Department of Ecology, Evolution and Environmental Biology, Columbia University, 1200 Amsterdam Avenue, New York, NY 10027, USA. Email: vioreldpopescu@gmail.com

^4^ School of Environmental Studies, University of Victoria, PO Box 1700 STN CSC, Victoria, BC V8W 2Y2 Canada. Email: marissadyck@uvic.ca

^5^ Department of Fish, Wildlife and Conservation Biology, Graduate Degree Program in Ecology, 1474 Campus Delivery, Colorado State University, Fort Collins, CO 80523, United States of America. Email: larissa.bailey@colostate.edu

*Corresponding author

Any use of trade, firm, or product names is for descriptive purposes only and does not imply endorsement by the U.S. Government.

**Supplemental Materials**

**
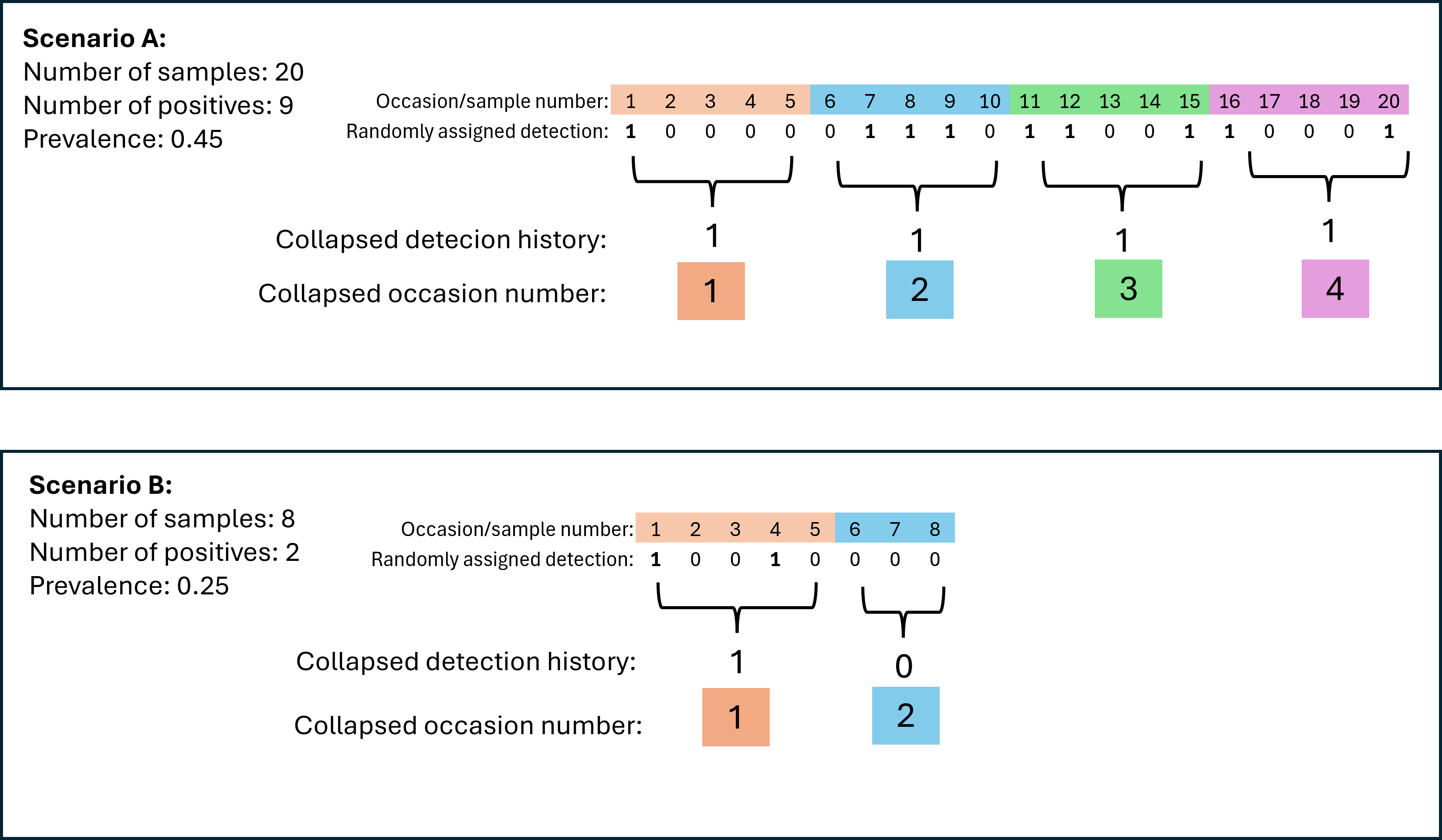
**

**Figure. S1** Schematic diagram illustrating the process for randomly assigning *Batrachochytrium dendrobatidis* (Bd) detection based on the total number of samples and total number of positives reported for each site. Randomization was necessary because dates of sampling were not reported. We further collapsed the capture histories by combining five occasions/samples into a single occasion to avoid computationally expensive model runs.

**Table S1.** Description of the six General Circulation Models representing different plausible future climate scenarios in the southern Rocky Mountains (Hegewisch and Abatzoglou, 2017).

| **Model** | **Representative future** | **Change in**  **Dec-Feb precip** | **Change in**  **Dec-Feb temp** |
| --- | --- | --- | --- |
| CanESM2 | Hot, wet winter | 0.8 | 6.947 |
| GFDL-ESM2M | Warm, wet winter | 0.2 | 3.635 |
| inmcm4 | Warm, dry winter | 0.3 | 3.722 |
| IPSL-CM5A-LR | Hot, dry winter | 0.2 | 4.886 |
| **Model** | **Representative future** | **Change in**  **Mar-May precip (inches)** | **Change in**  **Mar-May temp (^o^F)** |
| MIROC-ESM-CHEM | Hot, dry spring | 1.7 | 8.396 |
| HADGEM2-ES365 | Hot, wet spring | -0.6 | 6.376 |

***Climate Data Extraction***

We aggregated climate data from multiple sources for use in the hydroperiod and occupancy modeling. We downloaded historical (1985 – 2020) and future (2040 – 2069) 4-km resolution climate data for the boreal toad sites using packages *AOI* and *climateR* (Johnson, 2024, 2023) (Johnson 2023, 2024) in R v4.2.3 (R Core Team, 2024). The historical climate dataset contained daily surface meteorological data (gridMET,<https://www.climatologylab.org/gridmet.html>) from 1979 to present (Abatzoglou, 2013). The future climate data were from multivariate adaptative constructed analog datasets (MACA;<https://climate.northwestknowledge.net/MACA/index.php>) which downscale model outputs from general circulation models (GCMs) for two future Representative Concentration Pathways (RCPs) 4.5 and 8.5 (Abatzoglou and Brown, 2012). We selected six GCMs (HadGEM2-ES365, CanESM2, IPSL-CM5A-LR, GFDL-ESM2M, inmcm4, and MIROC-ESM-CHEM) that represent a range of plausible futures in the SRM (Table S1; Steen, 2017) for both RCPs. We selected four variables for use in the hydroperiod modeling: precipitation (*pr*), minimum temperature (*tmin*), maximum temperature (*tmax*), and vapor pressure deficit (*vpd*) (Table S2). We then aggregated the daily data for each variable into a single (mean) monthly value for historical data (between 1985 and 2020), as well as a monthly mean value for each future GCM, and RCP scenario (between 2040 and 2069).

*Water balance*

The historical and future water balance data were extracted from a 4-km resolution monthly water-balance model for 1950 to 2099 under RCP 4.5 and 8.5 (Alder and Hostetler, 2021). We extracted data for three variables, mean snow water equivalent (*snow*), potential evapotranspiration (*pet*), and soil moisture storage (*stor*) (Table S2) for both our historical time period (1985 to 2020) and future climate scenarios between 2040 and 2069. Snowmelt is likely to drive water levels for wetlands in the SRM (Mote et al., 2005), thus, we used the *snow* data from the month prior and the total amount of *snow* in the preceding winter as covariates in our hydroperiod models that estimated the monthly probability of drying and surface water area.

*Continuous heat-insolation load index*

We extracted the mean continuous heat-insolation load index (CHILI; Theobald et al., 2015) for each of the hydrologic unit code 12 (HUC12) watersheds containing the boreal toad breeding sites. CHILI is an extension of the heat load index developed by (McCune and Keon, (2002) and a proxy for the effects of insolation and topographic shading on evapotranspiration (Theobald et al. 2015). CHILI values range from 0 (very cool) to 255 (very warm). We used this as a static covariate in our hydroperiod model, hypothesizing that breeding sites with higher CHILI values are likely to dry at a faster rate.

*Topography*

We used the NASA Shuttle Radar Topography Mission (SRTM) 30-m resolution digital elevation model dataset (Earth Science Data Systems, 2025) to derive topography metrics. We extracted the elevation and calculated slope and aspect for each breeding site. We also derived the Topographic Ruggedness Index (TRI), which evaluates the amount of elevation difference between adjacent cells of a digital elevation model. We calculated mean TRI at the HUC12 watershed level and extracted the TRI value for each breeding site; TRI values below 150 represent terrain with low ruggedness, values between 150 and 500 represent moderately rugged terrain, and values >500 indicate highly rugged and topographically complex areas.

*Wetlands*

We used the US National Wetland Inventory (NWI, United States Fish and Wildlife Service, 2004) to extract the amount of wetland area and the proportion wetland area within HUC12 watersheds containing breeding sites. We used two categories of wetlands within the NWI dataset: 1) freshwater emergent wetland (NWI code PEM; Cowardin class = Palustrine Emergent), which includes herbaceous marsh, fen, swale and wet meadow wetland habitat, and 2) freshwater forested and shrub wetlands (NWI codes = PFO and PSS; Cowardin class = Palustrine forested and/or Palustrine shrub), which includes forested swamp, wetland shrub bog, and wetland habitat. We used these two categories only, as they represented the majority of wetland habitat within any given HUC12 watershed and we hypothesized that the total area and proportion of wetland area within a watershed would indicate the capacity of the watershed to retain water, complementary to the soil moisture storage metric.

**Table S2.** Names and descriptions of variables used for hydroperiod modeling.

| **Variable group** | **Variable name** | **Variable description** | **Units** | **Data source** | **Resolution** |
| --- | --- | --- | --- | --- | --- |
| *Climate* | *pr* | Mean precipitation | Millimeters | GridMET^1^ (historic)  MACA^2^ (future) | 4 km |
|  | *tmax* | Mean maximum near-surface air temperature | Kelvin | GridMET (historic)  MACA (future) | 4 km |
|  | *tmin* | Mean minimum near-surface air temperature | Kelvin | GridMET (historic)  MACA (future) | 4 km |
|  | *vpd* | Mean vapor pressure deficit | Kilopascals | GridMET (historic)  MACA (future) | 4 km |
| *Water balance* | *snow* | Mean snow water equivalent | Millimeters | MWBM^3^ (historic and future) | 4 km |
|  | *pet* | Potential evapotranspiration | Millimeters | MWBM (historic and future) | 4 km |
|  | *stor* | Soil moisture storage | Millimeters | MWBM (historic and future) | 4 km |
| *CHILI* | *CHILI* | Continuous heat-insolation load index | 0 (very cool) – 255 (very warm) | Theobald et al. 2015 | 10 m |
| *Topographic* | *aspect* | Aspect | Radians | NASA-SRTM^4^ | 30 m |
|  | *altitude* | Altitude | Meters | NASA-SRTM | 30 m |
|  | *slope* | Slope | Degrees | NASA-SRTM (derived) | 30 m |
|  | *TRI* | Topographic ruggedness index | <150 = low,  150-500 = moderate,  >500 = high | NASA-SRTM (derived) - HUC12 and site levels | 30 m |
| *Wetlands* | *emergent* | Emergent wetland | Square meters | US NWI^5^ – HUC12 level | Vector data |
|  | *forester* | Forested wetland | Square meters | US NWI – HUC12 level | Vector data |

^1^ Abatzoglou, 2013

^2^Abatzoglou and Brown, 2012

^3^Alder and Hostetler, 2021

^4^Earth Science Data Systems, 2025

^5^United States Fish and Wildlife Service, 2004

**Table S3.** Coefficient estimates for the top hurdle model predicting hydroperiod (probability of drying) and area of standing water. Std. Error = Standard Error

| **Variables** | **Mean Coefficient Estimate** | **Std. Error** | **Lower 95% Credible Interval** | **Upper 95% Credible Interval** | **R-hat** | **Effective Sample Size (posterior distribution)** |
| --- | --- | --- | --- | --- | --- | --- |
| **Gamma submodel (water area)** | | | | | | |
| Intercept | 6.75 | 0.04 | 6.67 | 6.82 | 1.00 | 1238 |
| Wetland area | 1.54 | 0.03 | 1.48 | 1.60 | 1.00 | 1257 |
| Precipitation (*pr*) | -0.01 | 0.01 | -0.03 | 0.02 | 1.00 | 1325 |
| Vapor Pressure Deficit (*vpd*) | 0.01 | 0.02 | -0.03 | 0.06 | 1.00 | 1459 |
| Water storage (*stor*) | 0.04 | 0.03 | -0.01 | 0.09 | 1.00 | 1595 |
| Snow previous winter | -0.02 | 0.04 | -0.11 | 0.06 | 1.00 | 1495 |
| Snow previous month (June) | 0.02 | 0.02 | -0.02 | 0.06 | 1.00 | 1463 |
| Watershed area | 0.07 | 0.04 | 0.00 | 0.15 | 1.00 | 1361 |
| Forested wetland | -0.04 | 0.04 | -0.11 | 0.03 | 1.01 | 1193 |
| Emergent wetland | -0.02 | 0.04 | -0.10 | 0.06 | 1.00 | 1183 |
| TRI (ruggedness) | 0.00 | 0.04 | -0.07 | 0.07 | 1.00 | 1169 |
| Aspect | 0.01 | 0.03 | -0.04 | 0.07 | 1.00 | 1077 |
| CHILI | 0.00 | 0.03 | -0.06 | 0.07 | 1.00 | 1355 |
|  |  |  |  |  |  |  |
| **Logistic submodel (probability of drying)** | | | | | | |
| Intercept | -2.51 | 0.32 | -3.21 | -1.89 | 1.00 | 1476 |
| Wetland area | -2.04 | 0.30 | -2.65 | -1.47 | 1.00 | 1493 |
| Precipitation (*pr*) | 0.29 | 0.12 | 0.05 | 0.51 | 1.00 | 1654 |
| Vapor Pressure Deficit (*vpd*) | 0.84 | 0.22 | 0.42 | 1.29 | 1.00 | 1534 |
| Water storage (*stor*) | 0.43 | 0.25 | -0.04 | 0.94 | 1.00 | 1315 |
| Snow previous winter | -0.48 | 0.38 | -1.23 | 0.24 | 1.00 | 1427 |
| Snow previous month (June) | -0.62 | 0.29 | -1.18 | -0.06 | 1.00 | 1508 |
| Watershed area | 0.31 | 0.31 | -0.30 | 0.92 | 1.00 | 1496 |
| Forested wetland | -0.06 | 0.28 | -0.60 | 0.48 | 1.00 | 1432 |
| Emergent wetland | 0.74 | 0.35 | 0.06 | 1.44 | 1.00 | 1350 |
| TRI (ruggedness) | -0.79 | 0.33 | -1.45 | -0.16 | 1.00 | 1599 |
| Aspect | -0.05 | 0.25 | -0.54 | 0.44 | 1.00 | 1240 |
| CHILI | -0.02 | 0.25 | -0.52 | 0.46 | 1.00 | 1325 |


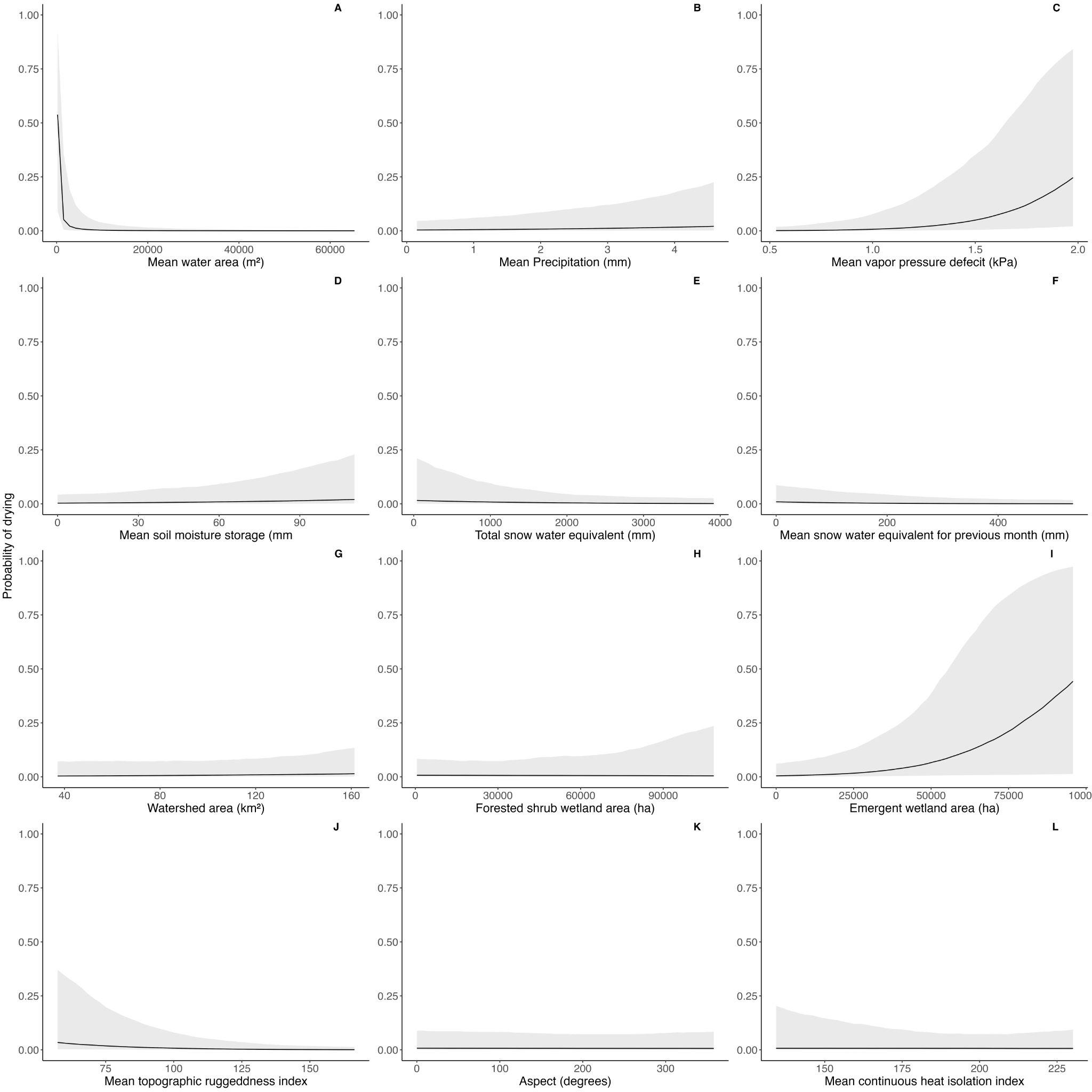


**Figure S2.** Marginal effects plots of coefficients included in the top hydroperiod model for the probability of drying.


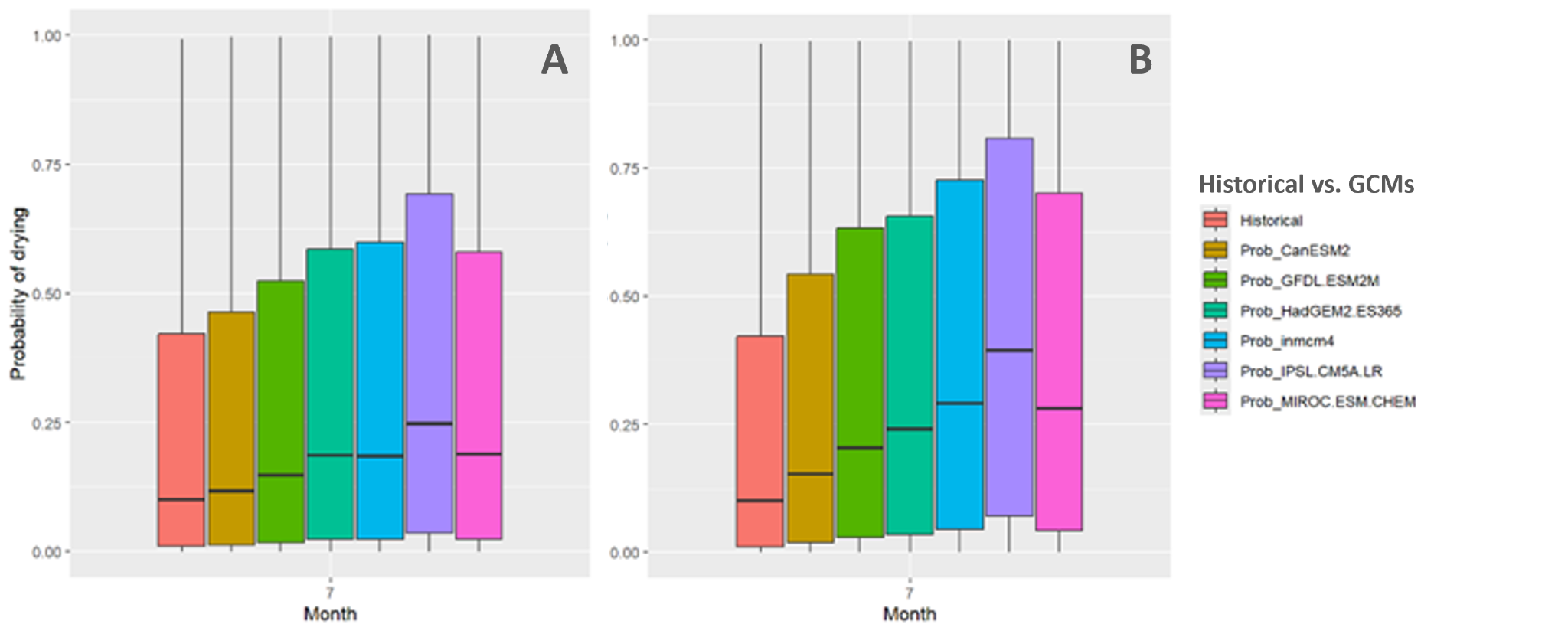


**Figure S3.** Change in probability of pond drying from historical to 2040-2069 under six Global Circulation Models (GCM) and two Representative Circulation Pathways (RCP): (a) RCP4.5 and (b) RCP8.5


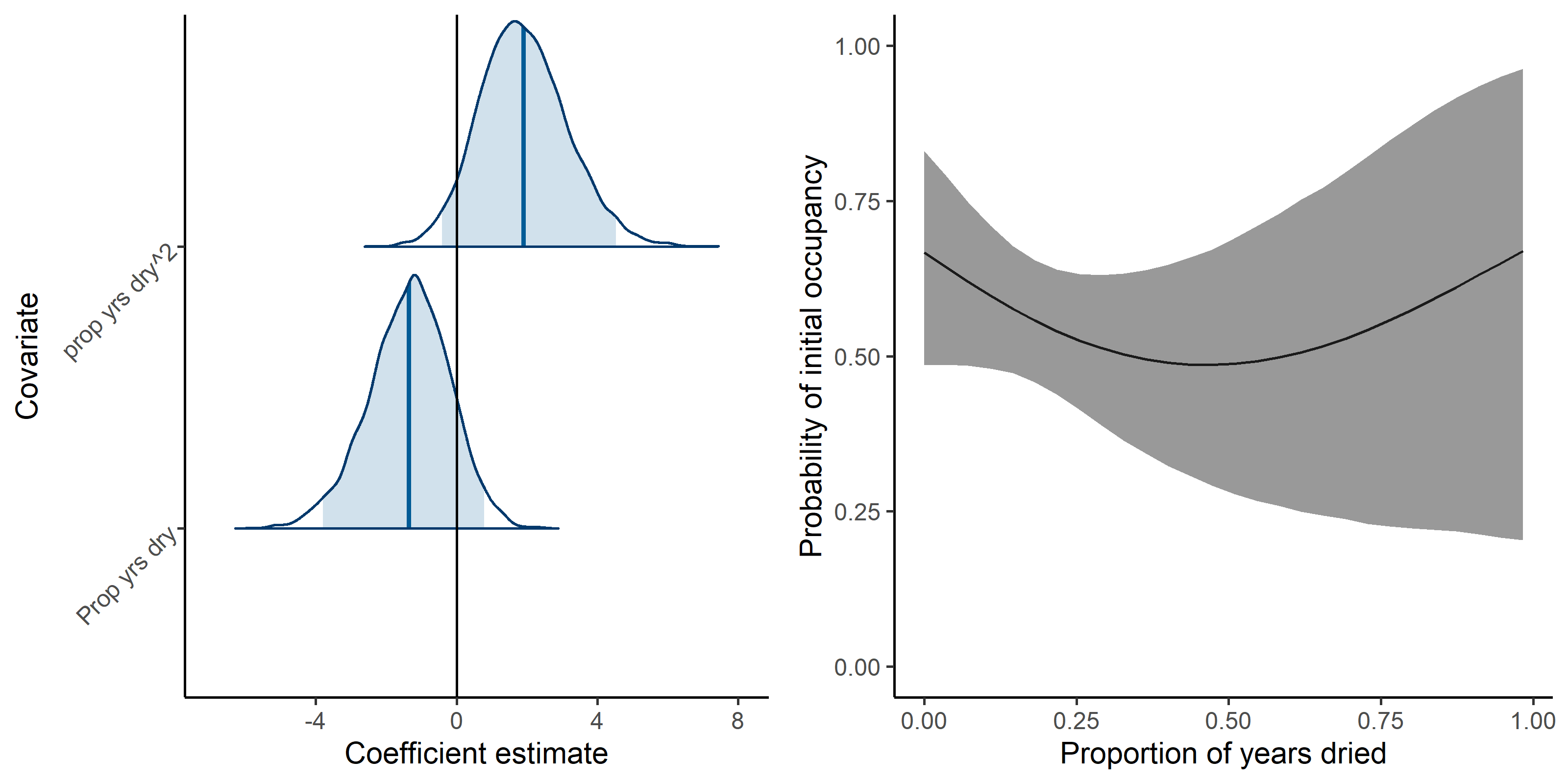


**b**

**a**

**b**

**a**

**Figure S4.** Plot of the posterior coefficient estimates for the effect of the quadratic term of the proportion of years dried on the probability of initial occupancy of breeding toads at a site (a) and associated marginal effects plot (b). In panel a, the thick blue line represents the median coefficient estimate and the shaded blue areas represent the 95% credible interval. In panel b, the solid black line represents the mean probability of occupancy for a range of values for the covariate, and the shaded gray area represents the 95% credible interval.


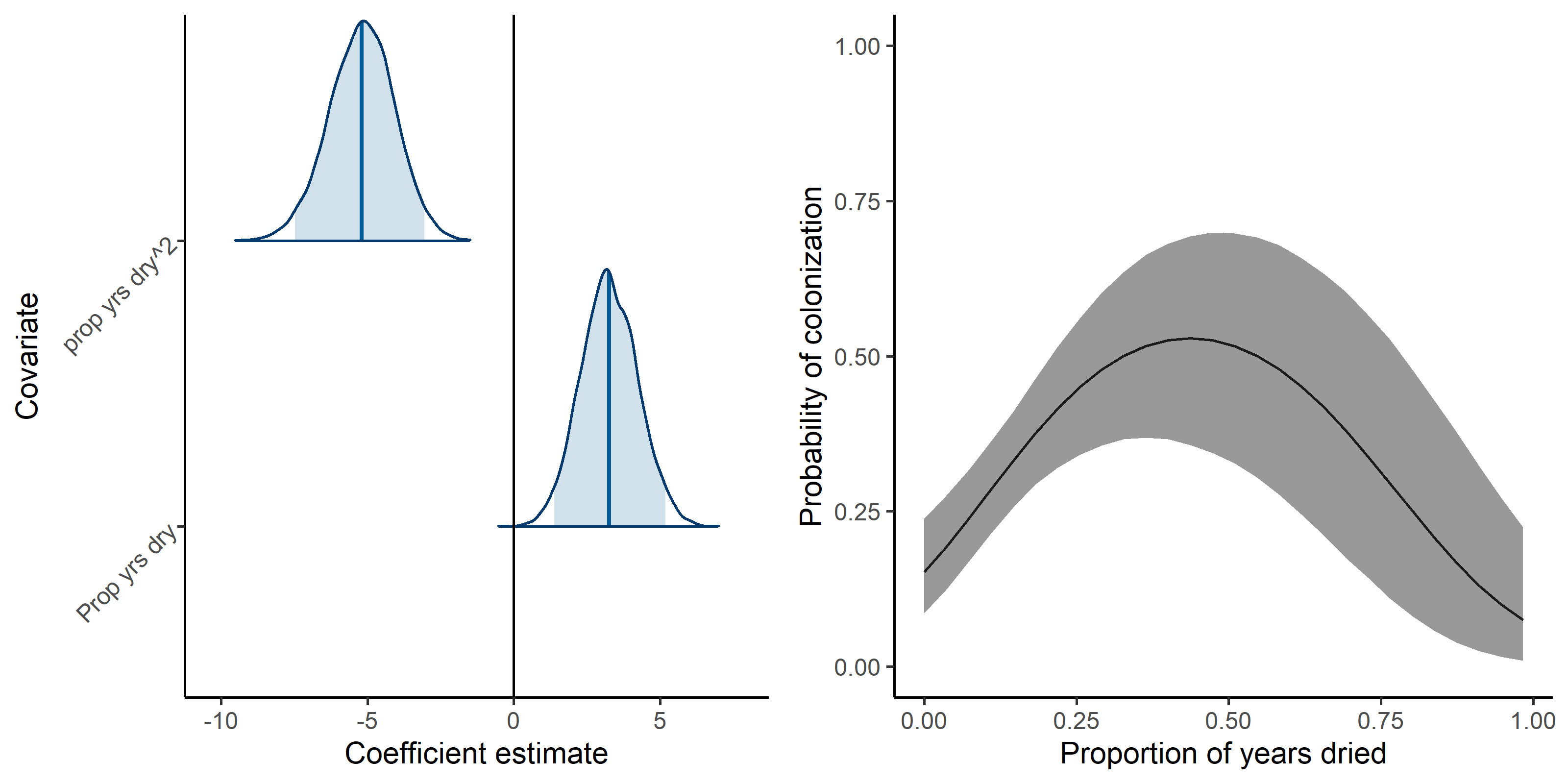


**a**

**b**

**a**

**b**

**Figure S5.** Plot of the posterior coefficient estimates for the effect of the quadratic term of the proportion of years dried on the probability of colonization of breeding toads at a site (a) and associated marginal effects plot (b). In panel a, the thick blue line represents the median coefficient estimate and the shaded blue areas represent the 95% credible interval. In panel b, the solid black line represents the mean probability of occupancy for a range of values for the covariate, and the shaded gray area represents the 95% credible interval.


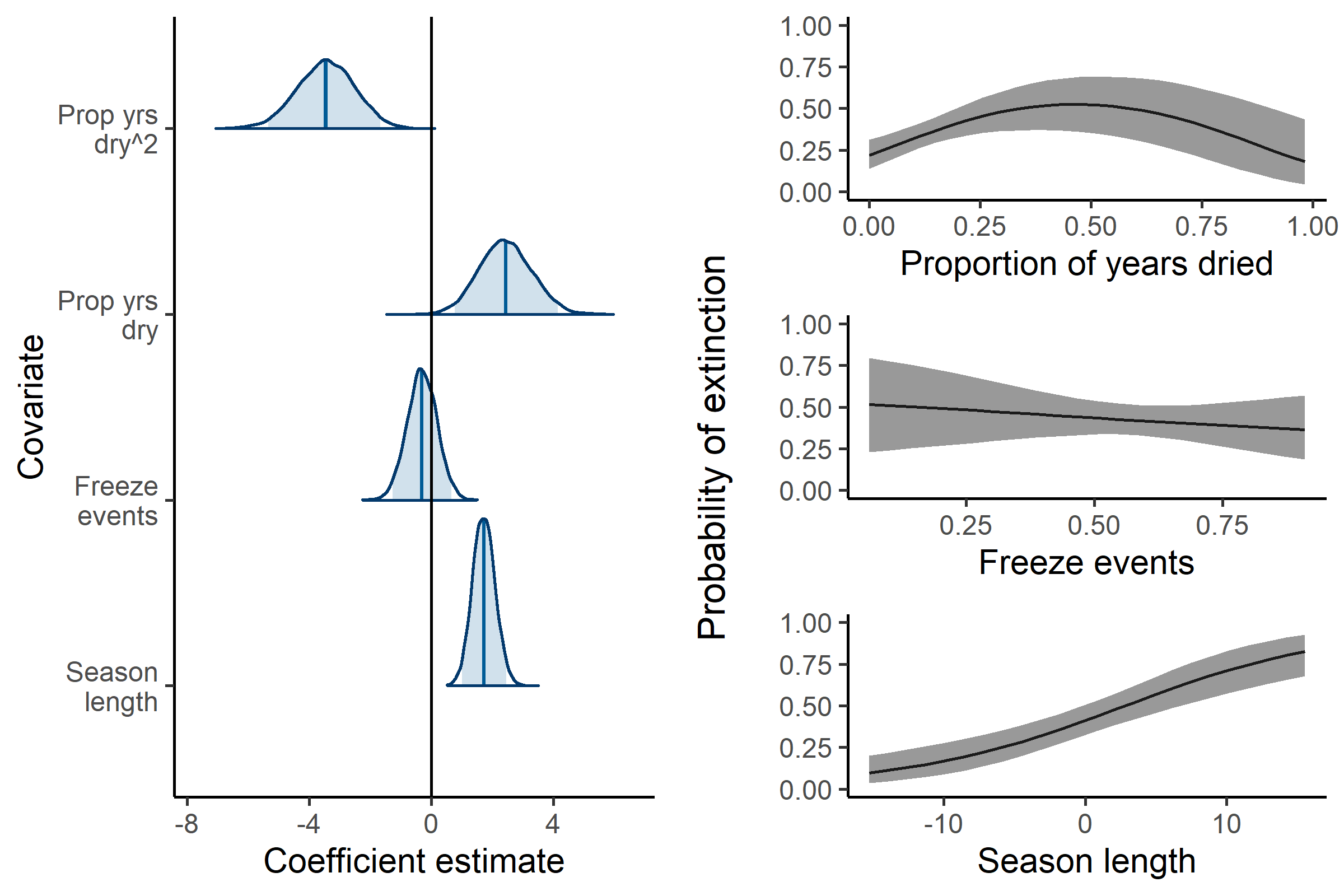


**b**

**d**

**c**

**a**

**b**

**d**

**c**

**a**

**Figure S6**. Plot of the posterior coefficient estimates for the effects of each covariate on the probability of extinction of breeding toads at a site (a) and associated marginal effects plot (b-d). In panel a, the thick blue line represents the median coefficient estimate and the shaded blue areas represent the 95% credible interval. In panels b-d, the solid black line represents the mean probability of occupancy for a range of values for the covariate, and the shaded gray area represents the 95% credible interval.


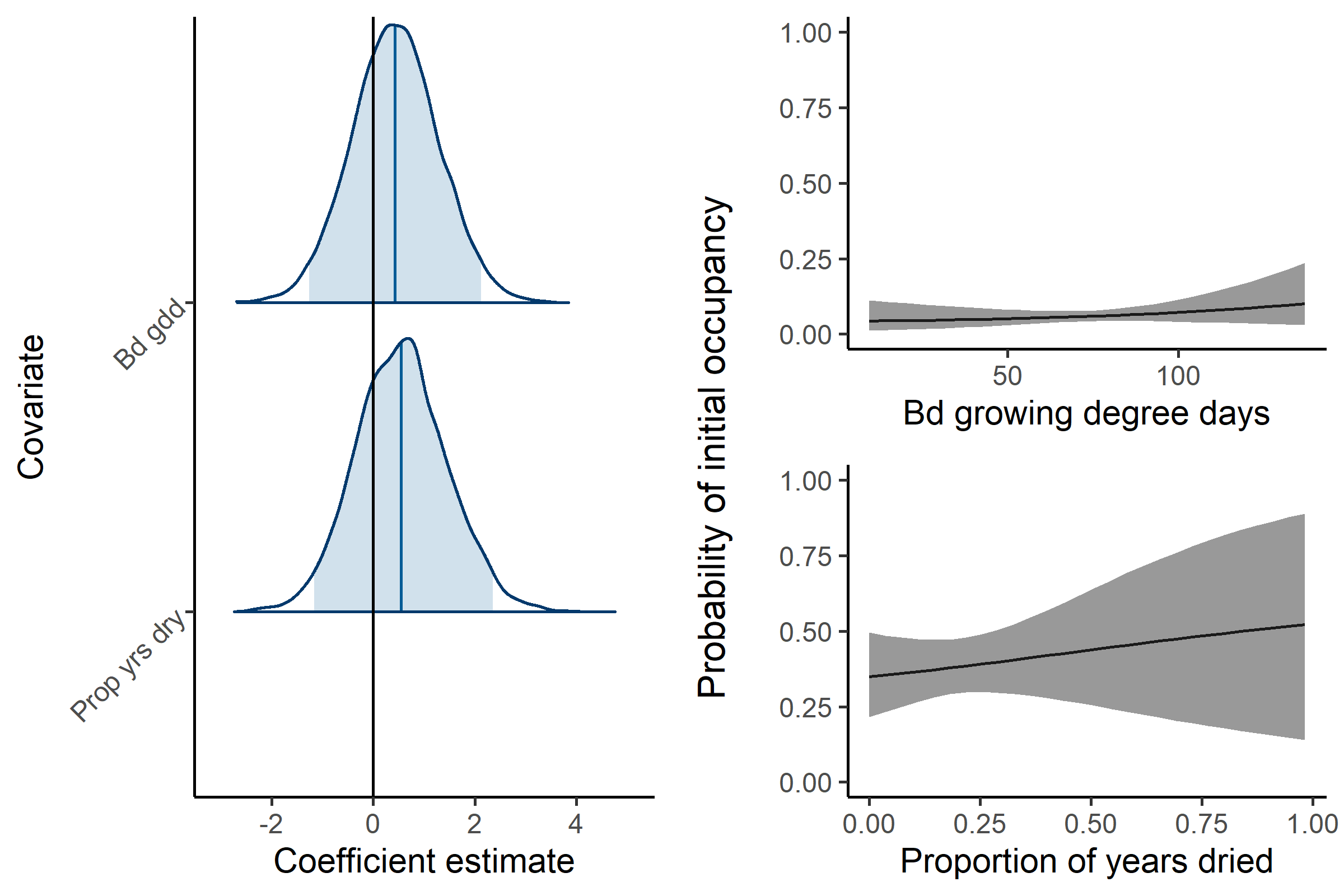


**c**

**b**

**a**

**c**

**b**

**a**

**Figure S7.** Plot of the posterior coefficient estimates for the effects of each covariate on the probability of initial occupancy of *Batrachochytrium dendrobatidis* (Bd) at a site (a) and associated marginal effects plot (b, c). In panel a, the thick blue line represents the median coefficient estimate and the shaded blue areas represent the 95% credible interval. In panels b and c, the solid black line represents the mean probability of occupancy for a range of values for the covariate, and the shaded gray area represents the 95% credible interval.


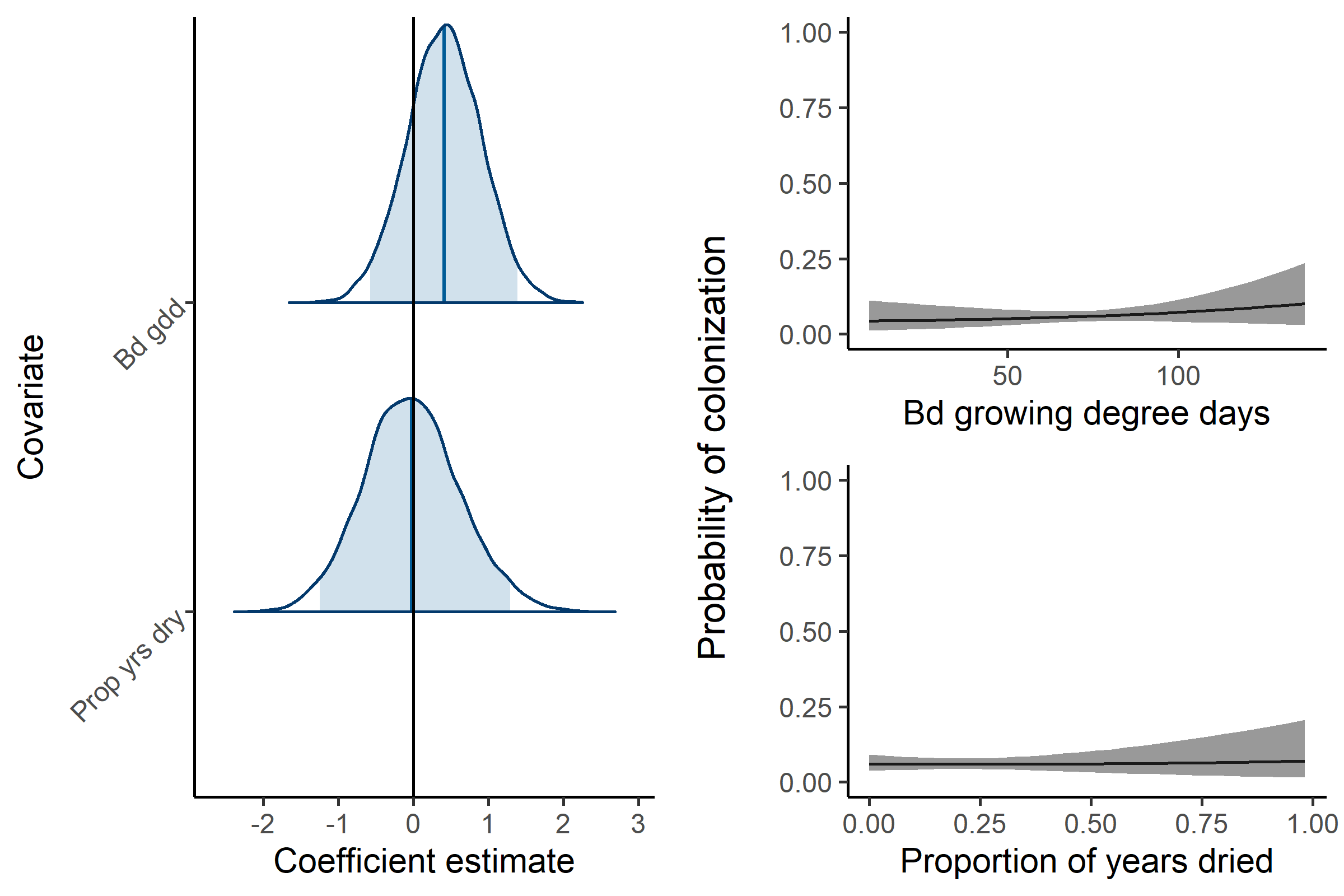


**c**

**b**

**a**

**c**

**b**

**a**

**Figure S8.** Plot of the posterior coefficient estimates for the effects of each covariate on the probability of colonization of *Batrachochytrium dendrobatidis* (Bd) at a site (a) and associated marginal effects plot (b, c). In panel a, the thick blue line represents the median coefficient estimate and the shaded blue areas represent the 95% credible interval. In panels b and c, the solid black line represents the mean probability of occupancy for a range of values for the covariate, and the shaded gray area represents the 95% credible interval.

**Table S4.** Comparison of mean occupancy values for each state under current and future climate scenarios for each mountain range containing boreal toad breeding sites in the southern Rocky Mountains. ‘Bd only’ refers to the state in which *Batrachochytrium dendrobatidis* is present but breeding toads are not. Representative Concentration Pathway = RCP.

| **Mountain range** | **Number of sites** | **Occupancy state** | **Current** | **RCP 4.5** | **RCP 8.5** |
| --- | --- | --- | --- | --- | --- |
| Elk & West Elk | 20 | Breeding toads, Bd free | 0.32 | 0.15 | 0.13 |
|  |  | Breeding toads, Bd present | 0.13 | 0.05 | 0.03 |
|  |  | Bd only | 0.09 | 0.11 | 0.12 |
| Elkhead | 3 | Breeding toads, Bd free | 0.19 | 0.11 | 0.09 |
|  |  | Breeding toads, Bd present | 0.07 | 0.04 | 0.03 |
|  |  | Bd only | 0.11 | 0.10 | 0.10 |
| Front Range | 45 | Breeding toads, Bd free | 0.29 | 0.16 | 0.14 |
|  |  | Breeding toads, Bd present | 0.12 | 0.06 | 0.05 |
|  |  | Bd only | 0.09 | 0.12 | 0.13 |
| Gore | 11 | Breeding toads, Bd free | 0.30 | 0.17 | 0.15 |
|  |  | Breeding toads, Bd present | 0.13 | 0.05 | 0.04 |
|  |  | Bd only | 0.08 | 0.10 | 0.10 |
| Grand Mesa | 1 | Breeding toads, Bd free | 0.38 | 0.13 | 0.11 |
|  |  | Breeding toads, Bd present | 0.16 | 0.05 | 0.04 |
|  |  | Bd only | 0.06 | 0.10 | 0.10 |
| Medicine Bow | 2 | Breeding toads, Bd free | 0.35 | 0.11 | 0.11 |
|  |  | Breeding toads, Bd present | 0.15 | 0.03 | 0.02 |
|  |  | Bd only | 0.07 | 0.13 | 0.13 |
| Mosquito & Ten-Mile | 10 | Breeding toads, Bd free | 0.34 | 0.21 | 0.18 |
|  |  | Breeding toads, Bd present | 0.14 | 0.08 | 0.06 |
|  |  | Bd only | 0.08 | 0.09 | 0.10 |
| Park & Sierra Madre | 6 | Breeding toads, Bd free | 0.23 | 0.15 | 0.14 |
|  |  | Breeding toads, Bd present | 0.10 | 0.05 | 0.04 |
|  |  | Bd only | 0.11 | 0.11 | 0.11 |
| San Juan | 8 | Breeding toads, Bd free | 0.26 | 0.12 | 0.11 |
|  |  | Breeding toads, Bd present | 0.11 | 0.04 | 0.03 |
|  |  | Bd only | 0.09 | 0.11 | 0.11 |
| Sawatch | 46 | Breeding toads, Bd free | 0.30 | 0.16 | 0.14 |
|  |  | Breeding toads, Bd present | 0.12 | 0.06 | 0.05 |
|  |  | Bd only | 0.08 | 0.11 | 0.12 |

Abatzoglou, J.T., 2013. Development of gridded surface meteorological data for ecological applications and modelling. International Journal of Climatology 33, 121–131. https://doi.org/10.1002/joc.3413

Abatzoglou, J.T., Brown, T.J., 2012. A comparison of statistical downscaling methods suited for wildfire applications. International Journal of Climatology 32, 772–780. https://doi.org/10.1002/joc.2312

Alder, J.R., Hostetler, S.W., 2021. CMIP5 MACAv2-METDATA monthly water balance model projections 1950-2099 for the contiguous United States. U.S. Geological Survey data release. https://doi.org/https://doi.org/10.5066/P9B2O22V

Earth Science Data Systems, N., 2025. NASA Shuttle Radar Topography Mission Global 1 arc second V003 | NASA Earthdata. https://www.earthdata.nasa.gov/data/catalog/lpcloud-srtmgl1-003

Hegewisch, K.C., Abatzoglou, J.T., 2017. “Future Climate Scatter” web tool. Climate Toolbox. https://climatetoolbox.org/

Johnson, M., 2024. climateR: climateR. v0.3.7. https://github.com/mikejohnson51/climateR

Johnson, M., 2023. AOI: Area of interest. v0.3.0. https://github.com/mikejohnson51/AOI/

McCune, B., Keon, D., 2002. Equations for potential annual direct incident radiation and heat load. Journal of Vegetation Science 13, 603–606. https://doi.org/10.1111/j.1654-1103.2002.tb02087.x

Mote, P.W., Hamlet, A.F., Clark, M.P., Lettenmaier, D.P., 2005. Declining mountain snowpack in Western North America. https://doi.org/10.1175/BAMS-86-1-39

R Core Team, 2024. R: A language and environment for statistical computing. Vienna, Austria

Steen, V., 2017. Projected Change in Climatically Relevant Variables for Boreal Toad. https://doi.org/10.5063/F1R78C42

Theobald, D.M., Harrison-Atlas, D., Monahan, W.B., Albano, C.M., 2015. Ecologically-Relevant Maps of Landforms and Physiographic Diversity for Climate Adaptation Planning. PLOS ONE 10, e0143619. https://doi.org/10.1371/journal.pone.0143619

United States Fish and Wildlife Service, 2004. National Wetlands Inventory. United States Fish and Wildlife Service. https://doi.org/https://doi.org/10.7944/USFWS.NWI
